# Research trends in neoadjuvant therapy for esophageal cancer: a bibliometric and meta-analysis

**DOI:** 10.3389/fimmu.2025.1646440

**Published:** 2025-11-19

**Authors:** Huaiyong Wang, Jian Zhang, Jiankun Yang, Hao Chang

**Affiliations:** Department of Thoracic Surgery, The First Affiliated Hospital of Harbin Medical University, Harbin, Heilongjiang, China

**Keywords:** esophageal cancer, neoadjuvant therapy, treatment regimens, immunotherapy, bibliometric analysis, meta-analysis

## Abstract

**Background:**

Neoadjuvant treatment followed by radical surgery has become the standard treatment approach for locally advanced esophageal cancer. We aimed to explore the development trends, research hotspots, and differences among treatment regimens in this field using bibliometric analysis and meta-analysis.

**Methods:**

Literature on neoadjuvant therapy for esophageal cancer was retrieved from PubMed, Embase, Cochrane Library, and Web of Science. Bibliometric analysis and visualization were conducted on publications since 2000 from Web of Science Core Collection (WoSCC) using CiteSpace, VOSviewer, and the bibliometrix package in RStudio. A meta-analysis of phase III randomized controlled trials (RCTs) involving different treatment regimens was performed using Stata/MP, based on studies screened from all four databases.

**Results:**

A total of 1,324 and 27 studies were included in the bibliometric analysis and meta-analysis, respectively. Overall, there was an increasing trend in the volume of publications in this field. The United States and the Karolinska Institute emerged as the leading country and institution in terms of publication volume. The most frequently cited journals and authors were Annals of Surgery and van Hagen P, respectively. Research hotspots have primarily focused on neoadjuvant chemotherapy (NCT) and chemoradiotherapy (NCRT), with a recent shift toward neoadjuvant immunotherapy (NIT). The pooled complete pathological response (pCR) rates were 0.08 for NCT, 0.29 for NCRT, 0.22 for neoadjuvant chemoimmunotherapy (NCIT), and 0.27 for NCRT combined with targeted therapy (NCRT+NTT). The pooled rates of tumor regression grade 1 (TRG1) were 0.09, 0.25, 0.30, and 0.37, respectively. The R0 resection rates were 0.87, 0.96, 0.99, and 0.96, while the incidence of grade ≥3 treatment-related adverse events (TRAEs) was 0.37, 0.66, 0.25, and 0.69, respectively.

**Conclusions:**

Neoadjuvant therapy for esophageal cancer has evolved significantly over the past decades. Recently, NIT has emerged as a key area of research interest. However, its clinical efficacy and safety require validation through long-term follow-up data from future phase III RCTs.

## 1 Introduction

Esophageal cancer is the malignant tumor with the seventh highest incidence worldwide and the sixth highest cancer-related mortality rate (1). Surgery combined with chemoradiotherapy or chemotherapy is the standard treatment for locally advanced resectable esophageal cancer (2). The JCOG9907 trial demonstrated that patients who received preoperative neoadjuvant therapy had longer overall survival (OS) than that of patients who underwent postoperative adjuvant therapy, without an increase in adverse events or surgical risks (3, 4). These findings support the inclusion of neoadjuvant therapy as an imperative ingredient in the treatment of locally advanced esophageal cancer.

Over the past 25 years, landmark studies, such as CROSS (5) and FLOT4 (6) have provided strong evidence regarding the efficacy of neoadjuvant chemoradiotherapy (NCRT) and neoadjuvant chemotherapy (NCT) in treating esophageal squamous cell carcinoma (ESCC) and esophageal adenocarcinoma (EAD), respectively. Recently, immune checkpoint inhibitors (ICIs) which target programmed cell death protein 1 and programmed death ligand 1 have made progress in the neoadjuvant treatment of pancreatic cancer. Favorable complete pathologic response (pCR) and major pathologic response (MPR) rates with acceptable levels of treatment-related adverse events (TRAEs) have also been reported in neoadjuvant therapy of esophageal cancer studies (7). Given the substantial evolution of neoadjuvant therapy for esophageal cancer, there is a growing need for a systematic bibliometric analysis of contributing countries, institutions, authors, and research keywords. However, bibliometric analysis alone is often insufficient to assess the comparative effectiveness and safety of different treatment regimens. Therefore, this study combined bibliometric analysis with a meta-analysis of phase III randomized controlled trials (RCTs), categorized by treatment regimens. This integrative approach enables the identification of research trends and emerging topics and facilitates direct comparisons of clinical outcomes across neoadjuvant strategies—providing a more comprehensive and integrated understanding of the current landscape in this field.

## 2 Materials and methods

### 2.1 Data source and search strategy

**Figure 1:**
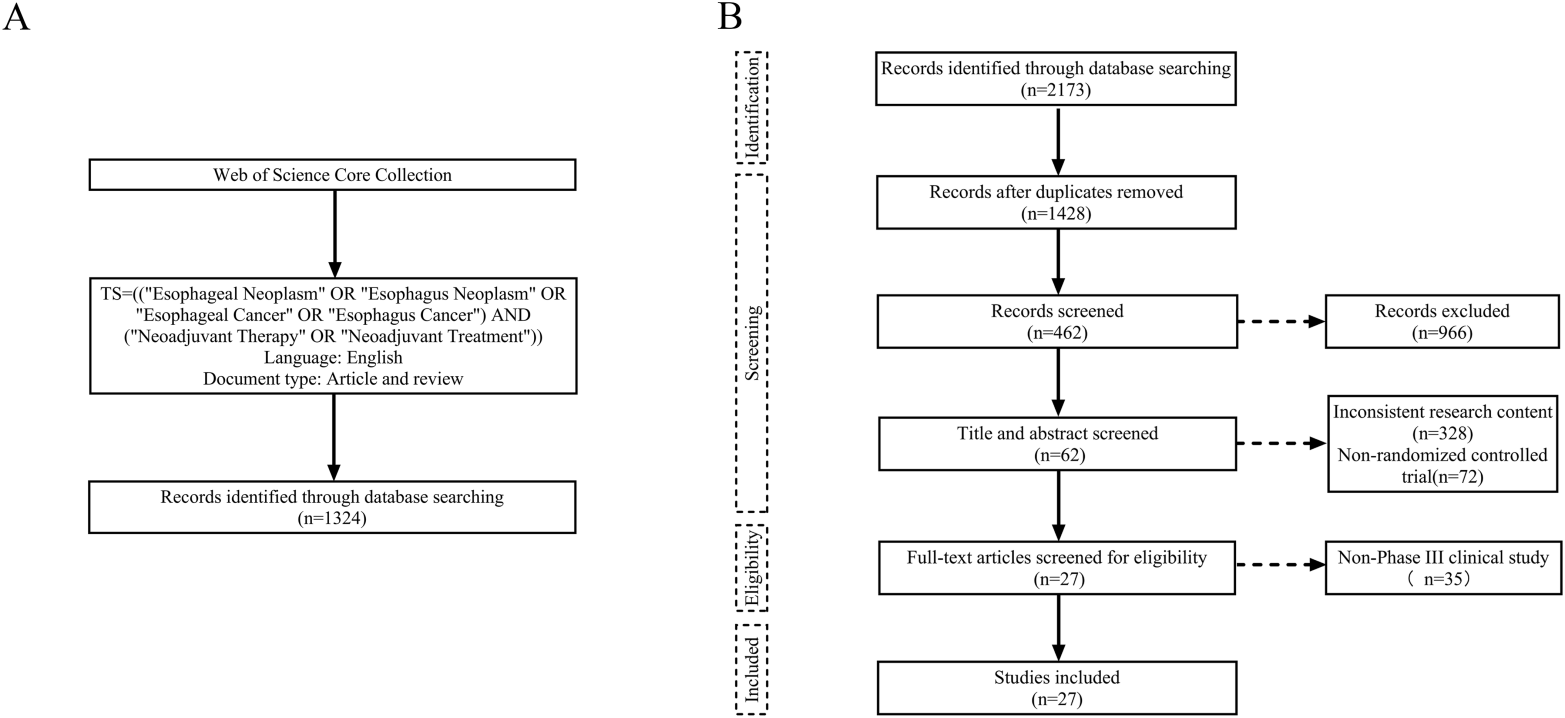
Flow charts of literature search and screening. **(A)** Bibliometric analysis. **(B)** Meta-analysis.

For the bibliometric analysis, literature records on neoadjuvant therapy for esophageal cancer published between January 2000 and May 2025 were searched from Web of Science Core Collection (WoSCC). And the search strategy used the following terms: TS=(“Esophageal Neoplasm” OR “Esophagus Neoplasm” OR “Esophageal Cancer” OR “Esophagus Cancer”) AND (“Neoadjuvant Therapy” OR “Neoadjuvant Treatment”), with results limited to articles and reviews published in English (**Figure 1A**).

For the meta-analysis, we conducted a comprehensive search in accordance with the Preferred Reporting Items for Systematic Reviews and Meta-Analyses (PRISMA statement). Studies were identified from four databases—PubMed, Embase, Cochrane Library, and Web of Science—with the final search completed in May 2025. The search formula was: (“Esophageal Neoplasm” OR “Esophagus Neoplasm” OR “Esophageal Cancer” OR “Esophagus Cancer”) AND (“Neoadjuvant Therapy” OR “Neoadjuvant Treatment”) AND (“Randomized controlled trial” OR “RCT”). Inclusion criteria were as follows: (1) pathologically confirmed stage I–IV esophageal cancer with the potential for surgical resection, (2) preoperative administration of neoadjuvant therapy, and (3) availability of complete patient clinical data, including pCR, tumor regression grade (TRG), and surgical outcomes. Exclusion criteria were as follows: (1) the primary endpoints unrelated to the efficacy of neoadjuvant therapy, (2) non-phase III RCTs, (3) incomplete or ongoing studies, (4) duplicate publications or overlapping data, (5) animal or cytological studies, reviews, case reports, and conference abstracts, and (6) non-English literatures. Two researchers independently performed the search and screening process, and any discrepancies were resolved through discussion to reach a consensus on the final list of included studies (**Figure 1B**).

### 2.2 Data extraction

In the bibliometric analysis, the retrieved records were exported as plain text files containing information such as the title, authors, institutions, countries, year of publication, abstract, keywords, references, DOI number, and publisher names. For the meta-analysis, two independent researchers separately extracted data from the included studies, covering: 1) article author(s), year of publication, and study identification number; 2) intent-to-treat population characteristics, including patient age, sex, pathological type, clinical tumor (T) and node (N) stages, and neoadjuvant treatment regimens; 3) key outcomes, such as pCR, TRG, grade ≥3 TRAEs, surgical resection rates, R0 resection rates, the incidence of surgical complications, and postoperative 30-day mortality.

### 2.3 Data analysis and quality evaluation

GraphPad Prism 10.1.2 (San Diego, CA, USA) was used to generate the study selection flowchart. CiteSpace 6.4.R1 was employed to visualize and analyze co-occurrence networks of authors, cited authors, institutions, countries, and references, as well as to cluster keywords. VOSviewer v1.6.20 was applied for the co-occurrence analysis of keywords and citations. Additionally, the bibliometrix package in RStudio (version 2024.12.1) was used to analyze changes in terms over time. Centrality was used as a metric to assess the influence and importance of academic entities within these networks.

**Figure 2:**
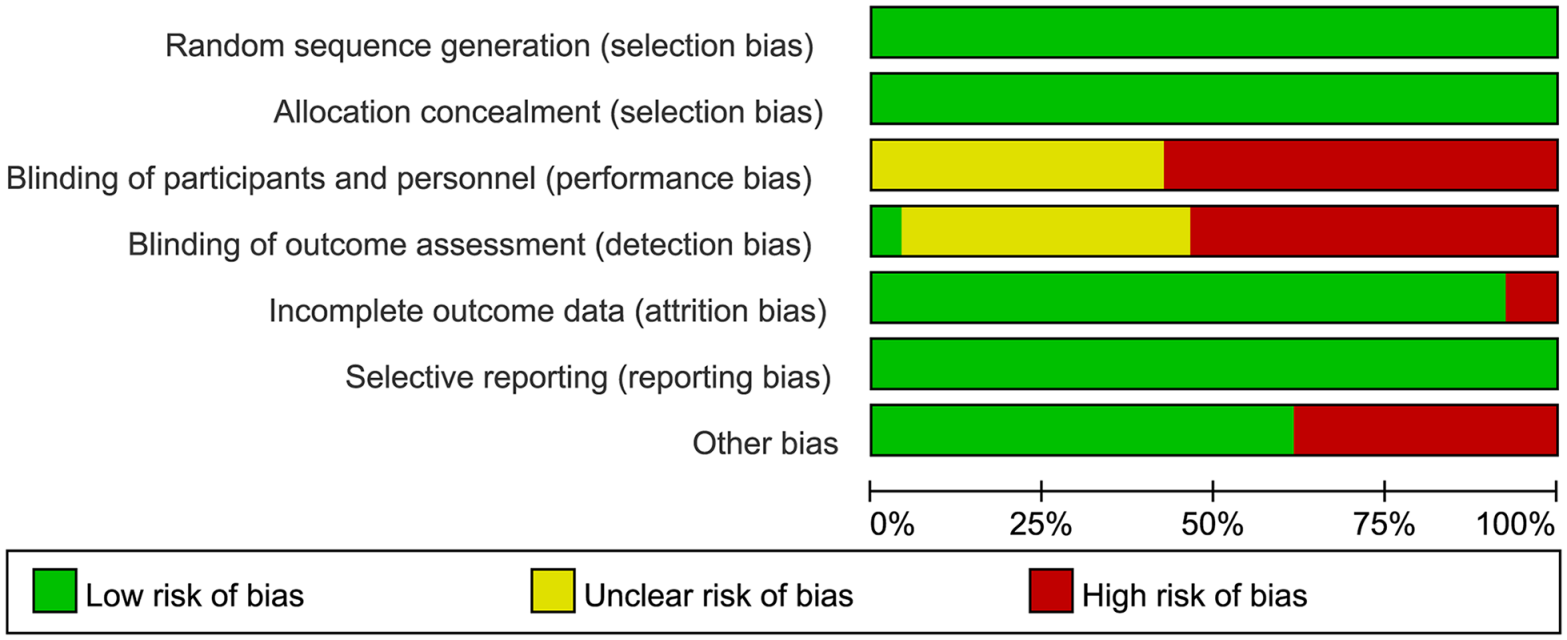
Summary of RCT literature quality assessment.

Data analysis for the meta-analysis was performed using Stata/MP 18.0. Heterogeneity was assessed using the I^2^ statistic and Cochran’s Q test. A fixed-effect model was applied if I^2^ was less than 50% and the P-value of the Q test was greater than 0.1; otherwise, a random-effects model was used. Sensitivity analyses were conducted to estimate the robustness of results and publication bias was evaluated visually with funnel plots and statistically using Egger’s test, with P-values less than 0.05 indicating significant bias. All of pooled effect sizes (ES) were presented using odds ratios (ORs) with 95% confidence intervals (CIs). The quality of RCTs was appraised using the Cochrane Collaboration’s Risk of Bias tool and visualized via Review Manager 5.4 (**Figure 2**, **Supplementary Figure S1**).

## 3 Results

### 3.1 Search results

**Figure 3:**
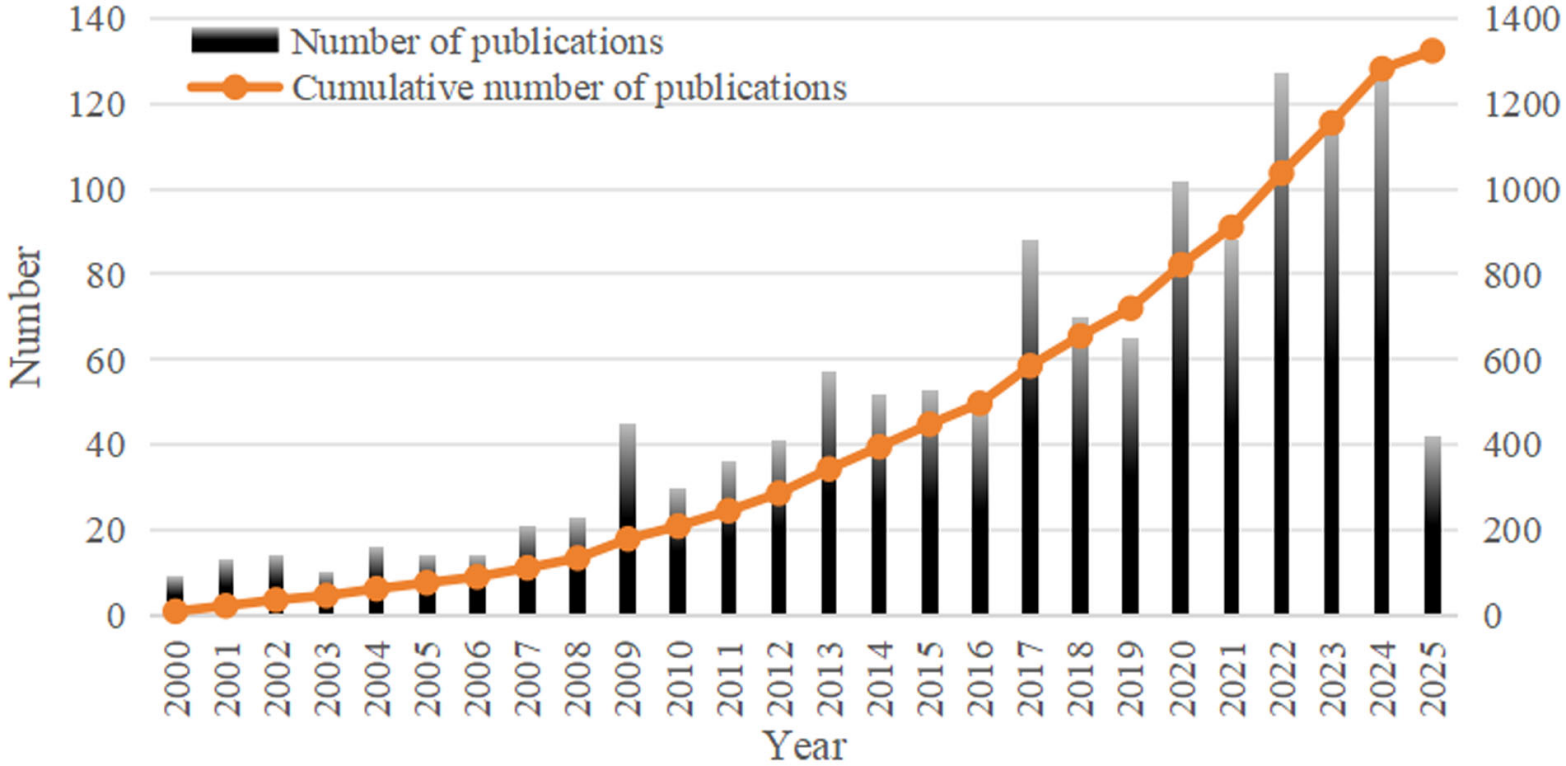
Annual and cumulative publications on neoadjuvant therapy for esophageal cancer.

With a total of 1,324 publications were retrieved for bibliometric analysis, comprising 1,137 original articles (85.88%) and 187 reviews (14.12%). From 2000 to 2024, the annual number of publications on neoadjuvant therapy for esophageal cancer gradually increased from 9 to 127, with the cumulative number also showing a clear upward trend (**Figure 3**). This growth reflects the increasing depth of research and highlights the field as a significant area of scientific interest.

For the meta-analysis, a total of 2,173 records were identified preliminarily. 27 publications met the inclusion criteria after deletion of duplicates and eligibility screening of titles, abstracts and full texts. These comprised 19 RCTs, including 10 studies on NCT, 14 on NCRT, 2 on NCIT, and 2 on NCRT combined with targeted therapy (NCRT+NTT) (**Table 1**, **Supplementary Table S1**).

**Table 1:** Summary of studies on neoadjuvant therapy for esophageal cancer.

Study	Study number	Clinical trial	Study type	ITT	Age	Male n[%]^a^	Pathological type	cT^b^	cN^b^	Neoadjuvant treatment regimen
Kato K, 2024 (8)	jRCTs031180202	JCOG1109	RCT	199	65.0	178 (89.4)	AD/SCC/BCC	T1-3	N0-3	DDP+FU
				202	64.0	178 (88.1)	AD/SCC/BCC	T1-3	N0-3	DDP+FU+DTX
				200	65.0	173 (86.5)	AD/SCC/BCC	T1-3	N0-3	DDP+FU+41.4 Gy
van Hagen P, 2012 (5) Shapiro J, 2015 (9) Eyck BM, 2021 (10)	NTR487	CROSS	RCT	178	60.0	134 (75.3)	AD/SCC/other	T1-3	N0-1	CBP+PTX+41.4 Gy
Hoeppner J, 2025 (11)	NCT02509286	ESOPEC	RCT	221	63.0	197 (89.1)	AD	T1-4	N0-1	FU+LV+OXP+DTX
				217	63.0	194 (89.4)	AD	T1-4	N0-1	CBP+PTX+41.4 Gy
Qin J, 2024 (12)	ChiCTR2000040034	ESCORT-NEO/NCCES01	RCT	132	63.0	116 (87.9)	SCC	T1b-3	N0-3	Cam+nab-PTX+DDP
				130	63.0	112 (86.2)	SCC	T1b-3	N0-3	Cam+PTX+DDP
				129	65.0	104 (80.6)	SCC	T1b-3	N0-3	PTX+DDP
Lee JL, 2004 (13)	NR	NR	RCT	51	63.0	46 (90.2)	SCC	NR	N0-1	DDP+FU+45.6 Gy
Ruhstaller T, 2018 (14)	NCT01107639	SAKK75/08	RCT	149	61.0	130 (87.2)	AD/SCC	T2-4a	N0/+	DDP+DTX+CET+45 Gy
				151	61.0	133 (88.1)	AD/SCC	T2-4a	N0/+	DDP+DTX+45 Gy
Tang H, 2023 (15) Wang H, 2021 (16)	NCT03001596	CMISG1701	RCT	132	NR	116 (87.9)	SCC	T3-4a	N0-1	PTX+DDP+40 Gy
				132	NR	110 (83.3)	SCC	T3-4a	N0-1	PTX+DDP
Mariette C, 2014 (17) Robb WB, 2015 (18) Robb WB, 2025 (19)	NCT00047112	FFCD 9901	RCT	98	58.1	87 (88.8)	AD/SCC/other	T1-3	N0-1	DDP+FU+45 Gy
Stahl M, 2005 (20)	NR	NR	RCT	86	57.0	69 (80.2)	SCC	T3-4	N0-1	FU+LV+ETO+DDP and ETO+DDP+40 Gy
Stahl M, 2009 (21) Stahl M, 2017 (22)	NR	POET	RCT	59	56.0	54 (91.5)	AD	T3-4	NR	FU+LV+DDP
				60	60.6	54 (90.0)	AD	T3-4	NR	FU+LV+DDP and ETO+DDP+30 Gy
Tepper J, 2008 (23)	NCT00003118	CALGB 9781	RCT	30	59.9	28 (93.3)	AD/SCC	T2-4	N0-1	DDP+FU+50.4 Gy
Yang H, 2018 (24) Yang H, 2021 (25)	NCT01216527	NEOCRTEC5010	RCT	224	56.0	190 (84.8)	SCC	T1-4	N0-1	NVB+DDP+40.0 Gy
Alderson D, 2017 (26)	NCT00041262	OE05	RCT	451	62.0	412 (91.4)	AD/SCC	T1-4	N0-1	DDP+FU
				446	62.0	398 (89.2)	AD/SCC	T1-4	N0-1	EPI+DDP+CAPE
Burmeister BH, 2005 (27)	NR	NR	RCT	128	61.0	106 (82.8)	AD/SCC	T1-3	N0-1	DDP+FU+35 Gy
Safran HP, 2022 (28)	NCT01196390	NRG Oncology/RTOG-1010	RCT	98	63.0	85 (86.7)	AD	T1-3	N0-2	PTX+CBP+TRA+50.4 Gy
				96	66.0	79 (82.3)	AD	T1-3	N0-2	PTX+CBP+50.4 Gy
Reynolds JV, 2023 (29)	NCT01726452	Neo-AEGIS	RCT	184	63.8	169 (91.8)	AD	T2-3	N0-3	EPI+DDP/OXP+FU/CAPE or FU+OXP+LV+DTX
				178	63.8	158 (88.8)	AD	T2-3	N0-3	PTX+CBP+41.4 Gy
Zheng Y, 2024 (30)	NCT04280822	HCHTOG 1909	RCT	127	66.0	97 (76.3)	SCC	T1b-3	N0/+	PTX+DDP+TOR
				125	68.0	97 (77.6)	SCC	T2-3	N0/+	PTX+DDP
Noronha V, 2025 (31)	CTRI/2014/04/004516	NR	RCT	210	55.0	132 (62.9)	SCC	T1-4	N0-3	DDP/CBP+FU
				210	56.0	121 (57.6)	SCC	T2-3	N0-3	DDP/CBP+PTX
Ando N, 2012 (32)	NCT00190554	JCOG9907	RCT	164	61.0	144 (87.8)	SCC	T1-3	N0-1	DDP+FU

a Data are n (%).

b Clinical T and N staging.

AD, adenocarcinoma; BCC, basal cell carcinoma; Cam, camrelizumab; CAPE, capecitabine; CBP, carboplatin; CET, cetuximab; DDP, cisplatin; DTX, docetaxel; EPI, epirubicin; ETO, etoposide; FU, fluorouracil; ITT, intention-to-treat population; LV, leucovorin; nab-PTX, nanoparticle albumin-bound paclitaxel; NR, not reported; NVB, vinorelbine; OXP, oxaliplatin; PTX, paclitaxel; RCT, randomized controlled trial; SCC, squamous cell carcinoma; TOR, toripalimab; TRA, trastuzumab.

### 3.2 Bibliometric analysis

#### 3.2.1 Countries/regions and institutions

**Figure 4:**
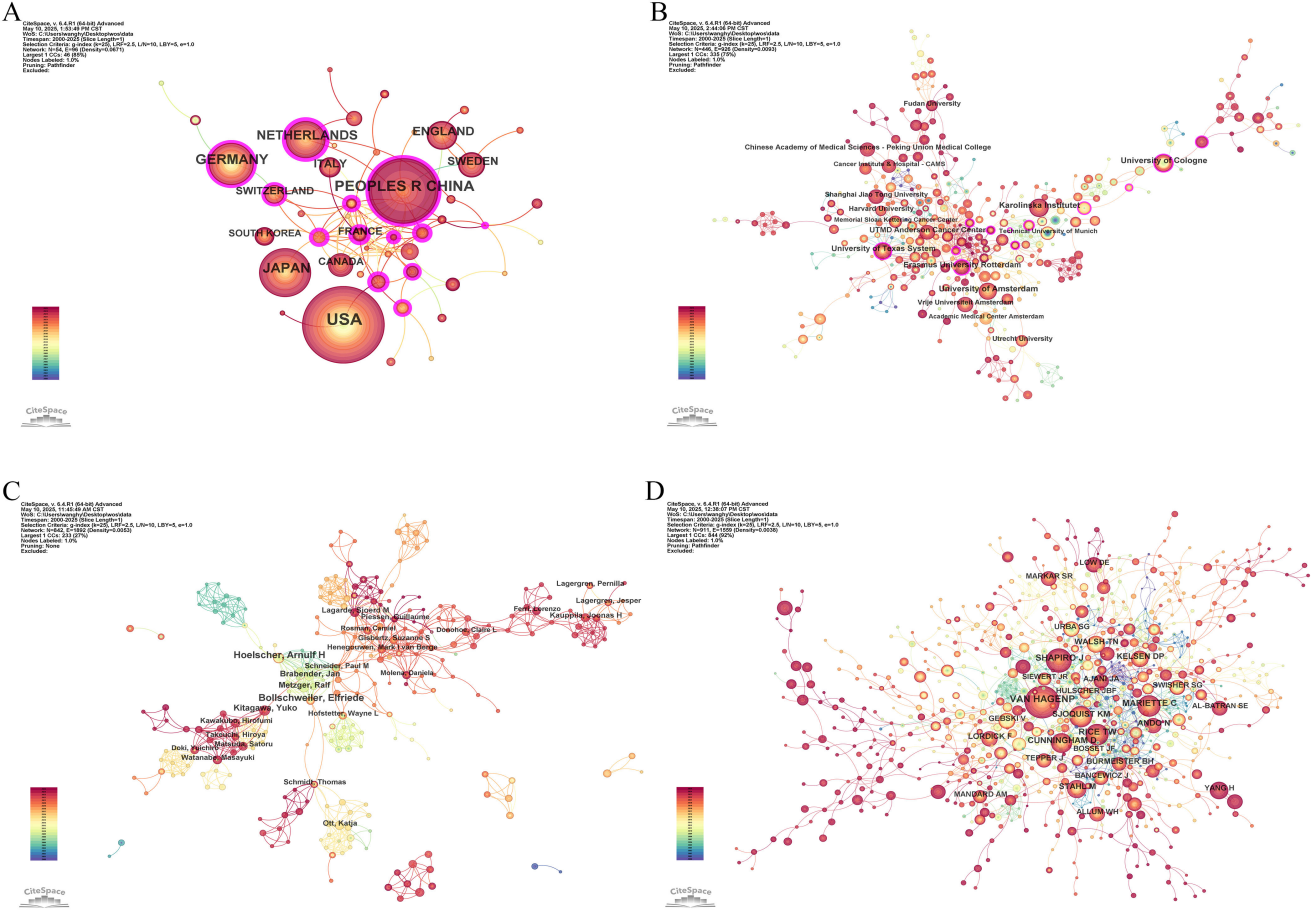
Co-occurrence network maps of neoadjuvant therapy for esophageal cancer. **(A)** Countries and regions. **(B)** Institutions. **(C)** Authors. **(D)** Cited authors.

54 countries and regions contributed to the literature on neoadjuvant therapy for esophageal cancer. The top five in terms of publication volume were the United States (370 publications, 27.95%), China (240, 18.13%), Germany (171, 12.92%), Japan (161, 12.16%), and the Netherlands (121, 9.14%). Among the top 10 publishing countries and regions, France had the highest centrality (0.32), suggesting that French research serves as an important intermediary and bridge in the global collaboration network. Notably, close research cooperation was observed among the United States, China, and several European countries (**Figure 4A**). In terms of citation metrics, the United States received significantly more citations than other countries, highlighting its leading role and strong academic influence in this field (**Table 2**).

**Table 2:** The top ten countries/regions in terms of publication volume and citations.

Rank	Country/region	Count	Centrality^a^	Rank	Cited country/region	Total citation	Average article citation
1	USA	370	0.06	1	USA	9137	30.00
2	China	240	0.17	2	Germany	4582	32.70
3	Germany	171	0.12	3	China	2955	11.70
4	Japan	161	0	4	Netherlands	2885	33.50
5	Netherlands	121	0.22	5	Japan	2656	17.80
6	England	88	0.08	6	United Kingdom	1360	29.60
7	Italy	59	0	7	France	926	38.60
8	Sweden	54	0.07	8	Italy	783	18.20
9	Switzerland	44	0.18	9	Korea	738	24.60
10	France	38	0.32	10	Ireland	731	34.80

a Centrality is related to importance and influence.

A total of 446 institutions contributed to the relevant literature. The top five institutions by number of publications were: Karolinska Institutet (51 publications, 3.85%), University of Amsterdam (48, 3.63%), University of Cologne (48, 3.63%), University of Texas System (44, 3.32%), and Erasmus University Rotterdam (41, 3.10%). Among the top 10 institutions, Erasmus University Rotterdam, University of Cologne, and University of Texas System exhibited the highest centrality scores, indicating their prominent roles in the collaborative research network (**Table 3**). Institutional collaboration and co-occurrence patterns are visualized in **Figure 4B**.

**Table 3:** The top ten institution in terms of publication volume.

Rank	Institution	Count	Centrality^a^	Year	Country/region
1	Karolinska Institutet	51	0.03	2006	Sweden
2	University of Amsterdam	48	0.06	2005	Netherlands
3	University of Cologne	48	0.17	2005	Germany
4	University of Texas System	44	0.15	2002	USA
5	Erasmus University Rotterdam	41	0.23	2008	Netherlands
6	UTMD Anderson Cancer Center	35	0.06	2007	USA
7	Chinese Academy of Medical Sciences - Peking Union Medical College	30	0.01	2018	China
8	Harvard University	29	0.08	2001	USA
9	Utrecht University	28	0.07	2011	Netherlands
10	Fudan University	26	0.09	2017	China

a Centrality is related to importance and influence.

#### 3.2.2 Authors and cited authors

In the analysis of contributing authors, Hoelscher Arnulf H. and Bollschweiler Elfriede were identified as particularly prolific, having published 21 and 19 articles, respectively. In terms of citation frequency, Van Hagen P. was the most cited author, with 478 citations (**Table 4**). Although Ajani JA. was not among the top 10 most cited authors, he exhibited the highest centrality score (0.14), indicating significant influence within the citation network. Visualizations of co-authorship and co-citation networks are presented in **Figures 4C, D**.

**Table 4:** The top ten authors in terms of publication volume and total citations.

Rank	Author	Count	Centrality^a^	Rank	Cited author	Citation	Centrality^a^
1	Hoelscher Arnulf H	21	0.02	1	van Hagen P	478	0.02
2	Bollschweiler Elfriede	19	0.04	2	Rice Thomas W	260	0.04
3	Kitagawa Yuko	13	0.04	3	Shapiro Joel	258	0.01
4	Metzger Ralf	12	0	4	Mariette Christophe	247	0.05
5	Brabender Jan	11	0	5	Cunningham David	224	0.03
6	Kauppila Joonas H	10	0	6	Sjoquist Katrin M	193	0.01
7	Lagarde Sjoerd M	10	0.01	7	Stahl Michael	192	0.02
8	Ott Katja	10	0.01	8	Walsh Thomas N	181	0.02
9	Kawakubo Hirofumi	9	0	9	Kelsen David P	179	0.05
10	Schneider Paul M	9	0.07	10	Ando Nobutoshi	165	0.11

a Centrality is related to importance and influence.

#### 3.2.3 Journals and cited journals

**Figure 5:**
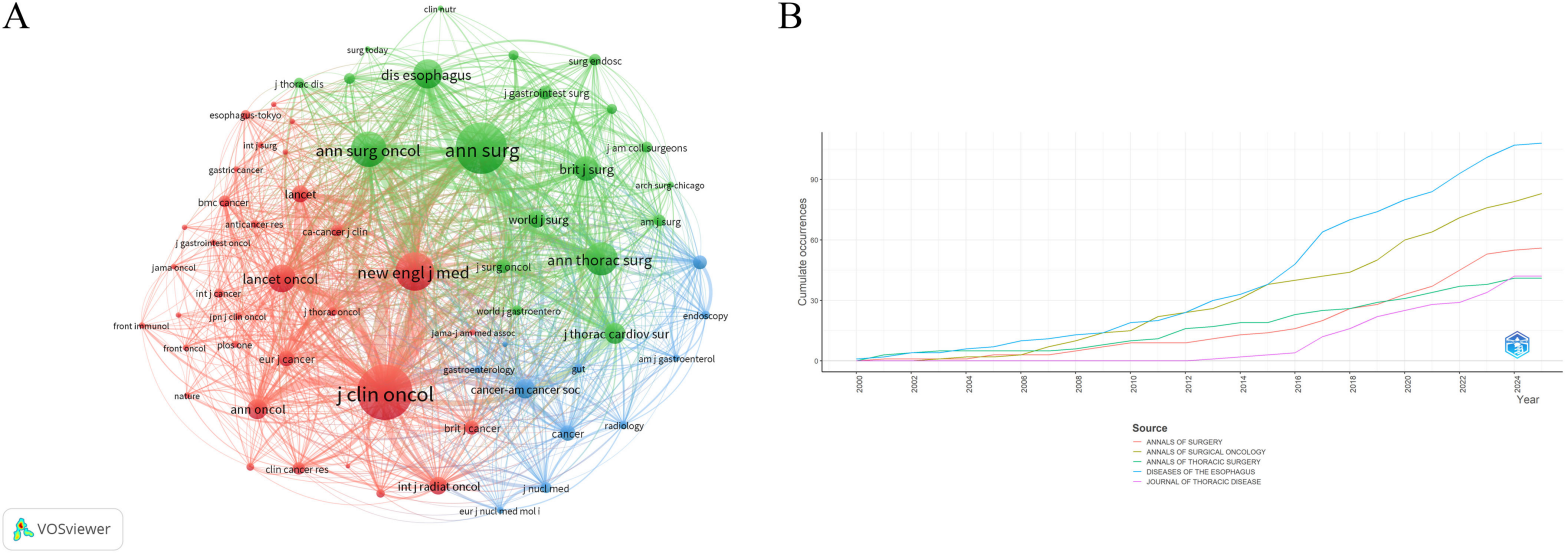
**(A)** Co-citation network map of journal. **(B)** Trends in cumulative journal publications.

A total of 749 journals were cited in the included literature. The five most frequently cited journals were: Annals of Surgery (903 citations), Journal of Clinical Oncology (889), New England Journal of Medicine (877), Annals of Surgical Oncology (808), and Diseases of The Esophagus (700). Notably, all of these journals are based in the United States, underscoring the country’s academic prominence in this research field (**Table 5**). A co-citation network visualization of the journals is shown in **Figure 5A**. Moreover, the number of published documents regarding neoadjuvant therapy for esophageal cancer has increased steadily over the past 25 years, with a marked acceleration after 2016. This trend likely reflects growing interest and advancements in neoadjuvant strategies followed by surgical intervention (**Figure 5B**).

**Table 5:** The top ten journal in terms of total citations.

Rank	Journal	Citation	Centrality^a^	Year	JCR^b^	IF	Country
1	Annals of Surgery	903	0	2000	1	7.9	USA
2	Journal of Clinical Oncology	889	0	2000	1	42.1	USA
3	New England Journal of Medicine	877	0.01	2000	1	96.3	USA
4	Annals of Surgical Oncology	808	0.02	2001	1	3.4	USA
5	Diseases of the Esophagus	700	0.01	2000	3	2.4	USA
6	Annals of Thoracic Surgery	690	0	2000	1	3.7	USA
7	Lancet Oncology	660	0	2006	1	41.6	England
8	British Journal of Surgery	547	0	2000	1	8.7	England
9	Journal of Thoracic and Cardiovascular Surgery	500	0	2000	1	4.9	USA
10	Cancer	487	0	2000	1	6.1	USA

a Centrality is related to importance and influence.

b JCR and IF according to the Journal Citation Reports 2024.

JCR, Journal Citation Reports; IF, impact factor.

#### 3.2.4 Analysis of references

**Figure 6:**
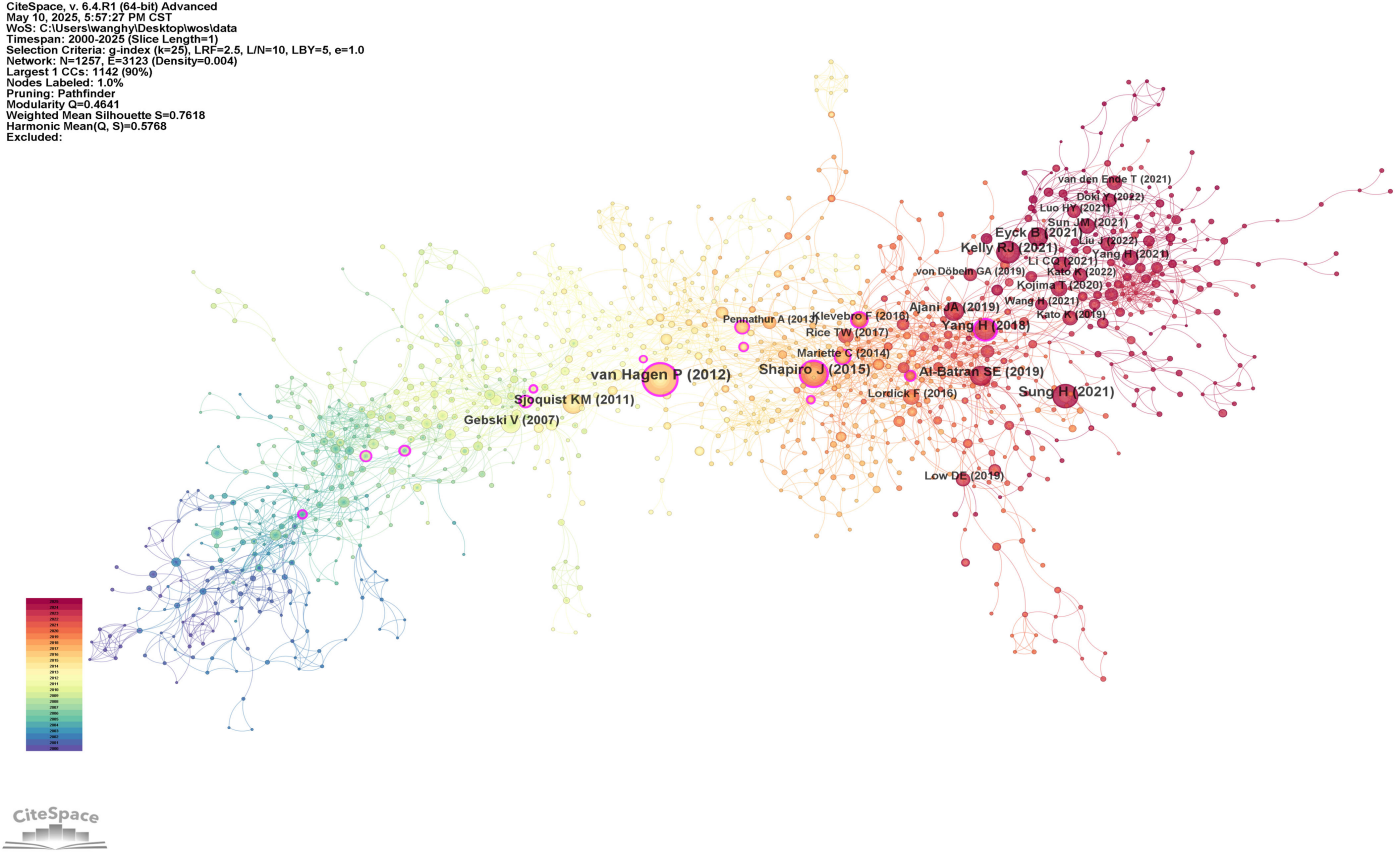
Co-occurrence network map of references on neoadjuvant therapy for esophageal cancer.

**Table 6** and **Figure 6** present the reference analysis. The CROSS study (5), a phase III RCT of NCRT for esophageal cancer, was the most frequently cited study, with 143 citations. This landmark study demonstrated that preoperative chemoradiotherapy significantly improves OS among patients with locally advanced esophageal cancer, providing foundational evidence that has shaped current treatment strategies. The remaining top five most cited references were authored by Sung et al. (1), Shapiro et al. (9), Kelly et al. (33), and Al-Batran et al. (6). Most of these highly cited works focus on NCRT in esophageal cancer treatment. However, it is noteworthy that the CheckMate 577 trial, reported by Kelly et al. and the CheckMate 577 Investigators (33), evaluated adjuvant nivolumab in resected esophageal cancer. Its high citation count suggests that immunotherapy is emerging as a significant research focus in this field.

**Table 6:** The top ten references in terms of total citations.

Rank	Article	First author	Year	Journal	Citation	Centrality^a^
1	Preoperative Chemoradiotherapy for Esophageal or Junctional Cancer	Van Hagen P	2012	New England Journal of Medicine	143	0.21
2	Global Cancer Statistics 2020: GLOBOCAN Estimates of Incidence and Mortality Worldwide for 36 Cancers in 185 Countries	Sung H	2021	Ca-A Cancer Journal for Clinicians	96	0.01
3	Neoadjuvant Chemoradiotherapy Plus Surgery Versus Surgery Alone for Oesophageal or Junctional Cancer (CROSS): Long-Term Results of A Randomised Controlled Trial	Shapiro J	2015	Lancet Oncology	92	0.27
4	Adjuvant Nivolumab in Resected Esophageal or Gastroesophageal Junction Cancer	Kelly RJ	2021	New England Journal of Medicine	79	0.06
5	Perioperative Chemotherapy with Fluorouracil Plus Leucovorin, Oxaliplatin, and Docetaxel Versus Fluorouracil or Capecitabine Plus Cisplatin and Epirubicin for Locally Advanced, Resectable Gastric or Gastro-Oesophageal Junction Adenocarcinoma (FLOT4): A Randomised, Phase 2/3 Trial	Al-Batran SE	2019	Lancet	72	0.02
6	Neoadjuvant Chemoradiotherapy Followed by Surgery Versus Surgery Alone for Locally Advanced Squamous Cell Carcinoma of the Esophagus (NEOCRTEC5010): A Phase III Multicenter, Randomized, Open-Label Clinical Trial	Yang H	2018	Journal of Clinical Oncology	68	0.23
7	Ten-Year Outcome of Neoadjuvant Chemoradiotherapy Plus Surgery for Esophageal Cancer: The Randomized Controlled CROSS Trial	Eyck B	2021	Journal of Clinical Oncology	66	0.02
8	Survival After Neoadjuvant Chemotherapy or Chemoradiotherapy for Resectable Oesophageal Carcinoma: An Updated Meta-Analysis	Sjoquist KM	2011	Lancet Oncology	65	0.02
9	Survival Benefits from Neoadjuvant Chemoradiotherapy or Chemotherapy in Oesophageal Carcinoma: A Meta-Analysis	Gebski V	2007	Lancet Oncology	56	0.01
10	Esophageal and Esophagogastric Junction Cancers, Version 2.2019, NCCN Clinical Practice Guidelines in Oncology	Ajani JA	2019	Journal of the National Comprehensive Cancer Network	56	0.03

a Centrality is related to importance and influence.

#### 3.2.5 Keywords and terms

**Figure 7:**
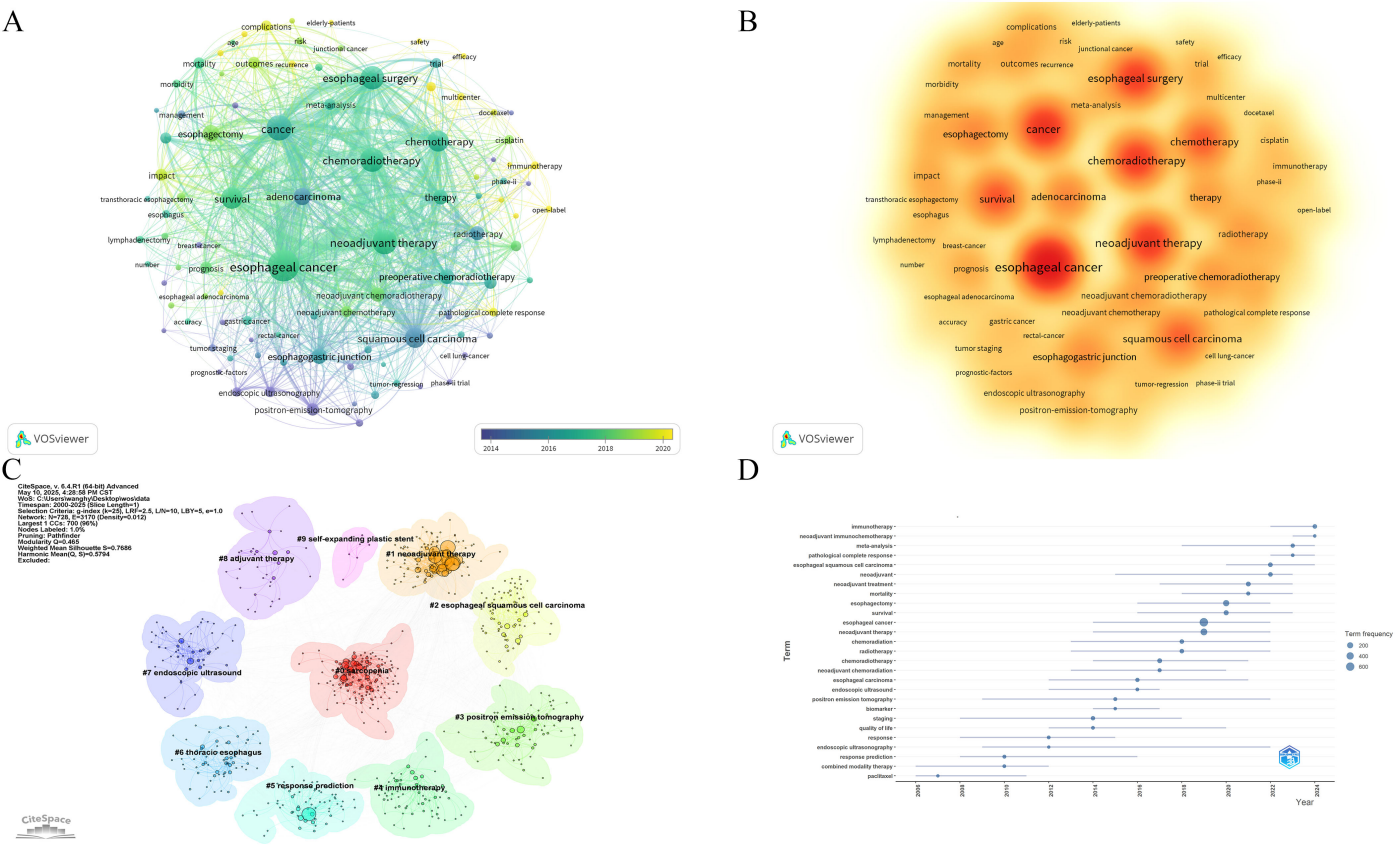
**(A)** Co-occurrence network map of keywords. **(B)** Hotspots map of keywords. **(C)** Clustering analysis chart of keywords. **(D)** Trends in terms based on keywords.

The 10 most frequently occurring keywords identified were: esophageal cancer, cancer, neoadjuvant therapy, esophageal surgery, chemoradiotherapy, squamous cell carcinoma, survival, chemotherapy, adenocarcinoma, and esophagogastric junction (**Table 7**). The frequency of these keywords indicates that chemoradiotherapy and chemotherapy have been the primary focus of research over the past 25 years. Keyword co-occurrence patterns and research hotspots are visualized in **Figures 7A, B**. Further clustering analysis of keywords revealed three main thematic areas: treatment regimens and methods (e.g., #1 neoadjuvant therapy, #4 immunotherapy, #8 adjuvant therapy, and #9 self-expanding plastic stent), and diagnostic techniques and treatment evaluation (e.g., #3 positron emission tomography, #5 response prediction, and #7 endoscopic ultrasound) (**Figure 7C**).

**Table 7:** The ten most frequently occurring keywords.

Rank	Keyword	Count	Centrality^a^	Year
1	esophageal cancer	849	0	2000
2	cancer	524	0	2000
3	neoadjuvant therapy	454	0.02	2001
4	esophageal surgery	423	0.01	2000
5	chemoradiotherapy	420	0.03	2000
6	squamous cell carcinoma	330	0.03	2000
7	survival	302	0.02	2003
8	chemotherapy	285	0.04	2000
9	adenocarcinoma	219	0.04	2000
10	esophagogastric junction	193	0.05	2001

a Centrality is related to importance and influence.

Additionally, term analysis based on keyword trends showed a clear shift in research focus—from traditional NCT and neoadjuvant radiotherapy to newer approaches, such as neoadjuvant immunotherapy (NIT) and NCIT (**Figure 7D**).

### 3.3 Meta-analysis

#### 3.3.1 Effectiveness of treatment

**Figure 8:**
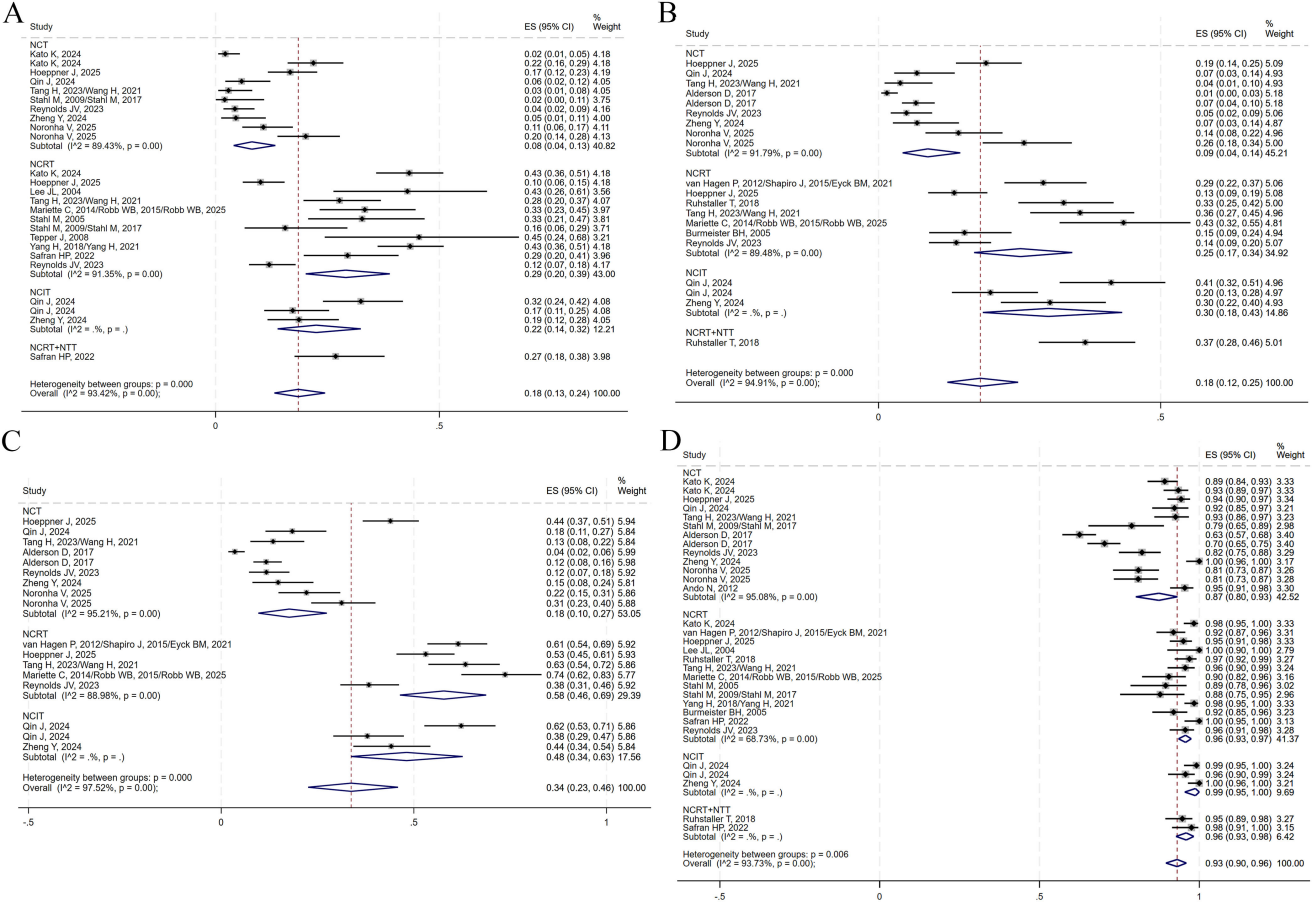
Forest plots on the efficacy of neoadjuvant therapy. **(A)** pCR. **(B)** TRG1. **(C)** TRG1 + 2. **(D)** R0 resection.

The presence of no residual live malignant cells in the primary tumor and the lymph nodes is defined as pCR. (i.e., ypT0N0cM0). A total of 14 studies reported pCR data. TRG was assessed using the Mandard scoring system, where TRG1 indicates no residual tumor cells and TRG2 indicates minimal residual tumor. TRG1 and combined TRG1 + 2 were reported in 11 and 9 studies, respectively. Notably, no studies in the NCRT+NTT group reported TRG1 + 2. The R0 resection rate, an important surgical outcome indicating complete tumor removal with negative margins, was reported in 18 studies. A random-effects model was applied to all analyses to account for substantial heterogeneity among studies. The pooled ES for pCR in the NCT, NCRT, NCIT, and NCRT+NTT groups were 0.08 (95% CI: 0.04–0.13; I²=89.43%), 0.29 (95% CI: 0.20–0.39; I²=91.35%), 0.22 (95% CI: 0.14–0.32), and 0.27 (95% CI: 0.18–0.38), respectively (**Figure 8A**). For TRG1, the ES values were 0.09 (95% CI: 0.04–0.14; I² = 91.79%) for NCT, 0.25 (95% CI: 0.17–0.34; I² = 89.48%) for NCRT, 0.30 (95% CI: 0.18–0.43) for NCIT, and 0.37 (95% CI: 0.28–0.46) for NCRT+NTT (**Figure 8B**). For TRG1 + 2, the ES was 0.18 (95% CI: 0.10–0.27; I²=95.21%) for NCT, 0.58 (95% CI: 0.46–0.69; I²=88.98%) for NCRT, and 0.48 (95% CI: 0.34–0.63) for NCIT (**Figure 8C**). The ES for R0 resection was 0.87 (95% CI: 0.80–0.93; I²=95.08%) for NCT, 0.96 (95% CI: 0.93–0.97; I²=68.73%) for NCRT, 0.99 (95% CI: 0.95–1.00) for NCIT, and 0.96 (95% CI: 0.93–0.98) for NCRT+NTT (**Figure 8D**).

#### 3.3.2 Surgery and safety

**Figure 9:**
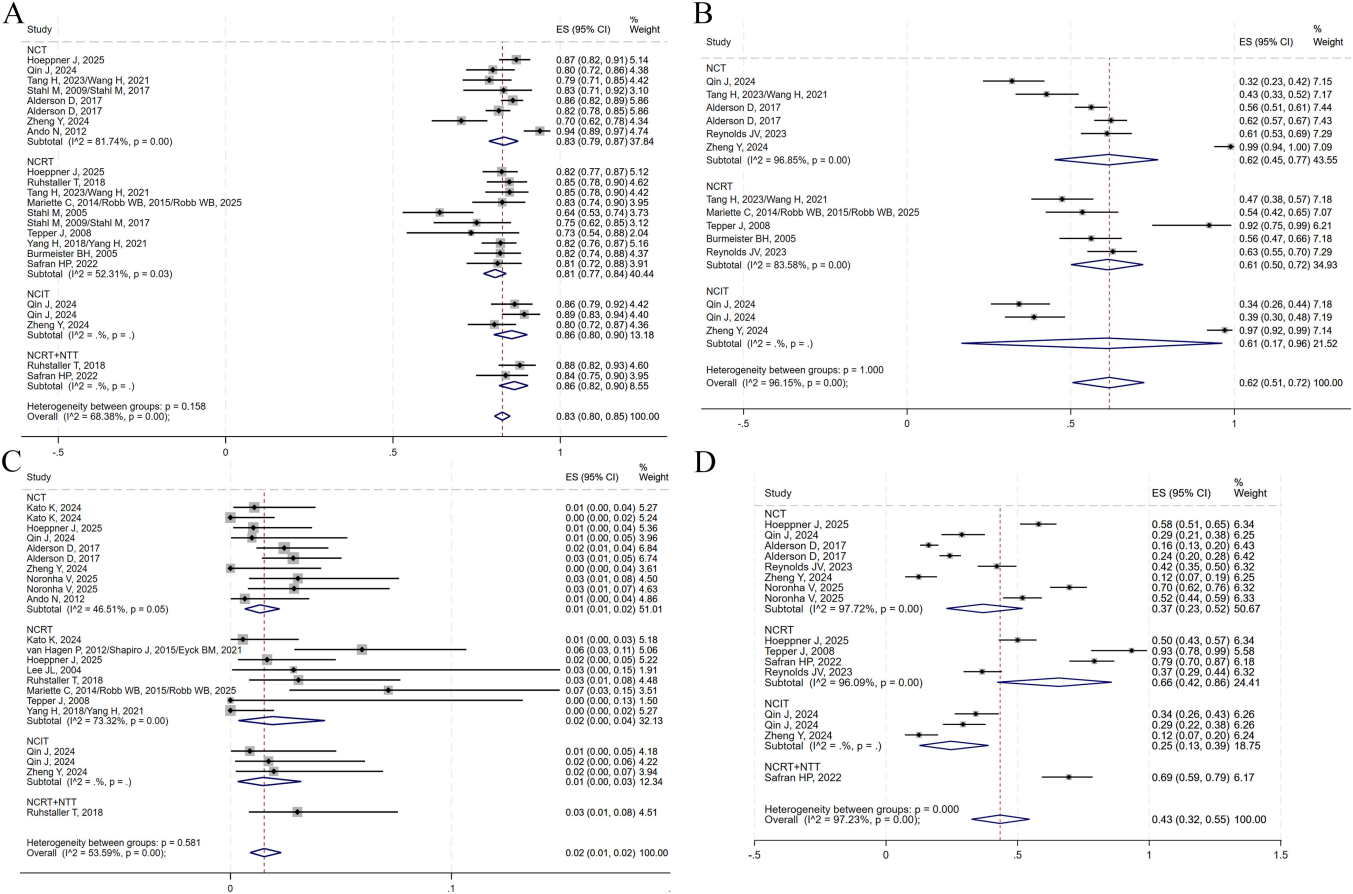
Forest plots on the neoadjuvant therapy. **(A)** Surgical resection. **(B)** Surgical complications. **(C)** Postoperative 30-day death. **(D)** TRAEs of grade ≥3.

A total of 14, 9, and 13 studies reported on surgical resection, surgical complications, and postoperative 30-day mortality, respectively. No data on surgical complication rates were available for the NCRT+NTT group. All analyses were conducted using a random-effects model. The pooled ES values for surgical resection in the NCT, NCRT, NCIT, and NCRT+NTT groups were 0.83 (95% CI: 0.79–0.87; I²=81.74%), 0.81 (95% CI: 0.77–0.84; I²=52.31%), 0.86 (95% CI: 0.80–0.90), and 0.86 (95% CI: 0.82–0.90), respectively (**Figure 9A**). For surgical complications, the ES was 0.62 (95% CI: 0.45–0.77; I²=96.85%) for NCT, 0.61 (95% CI: 0.50–0.72; I²=83.58%) for NCRT, and 0.61 (95% CI: 0.17–0.96) for NCIT (**Figure 9B**). For postoperative 30-day mortality, the ES was 0.01 (95% CI: 0.01–0.02; I²=46.51%) for NCT, 0.02 (95% CI: 0.00–0.04; I²=73.32%) for NCRT, 0.01 (95% CI: 0.00–0.03) for NCIT, and 0.03 (95% CI: 0.01–0.08) for NCRT+NTT (**Figure 9C**).

Additionally, eight studies reported on TRAEs of grade ≥3. Using a random-effects model, the ES for grade ≥3 TRAEs was 0.37 (95% CI: 0.23–0.52; I²=97.72%) for NCT, 0.66 (95% CI: 0.42–0.86; I²=96.09%) for NCRT, 0.25 (95% CI: 0.13–0.39) for NCIT, and 0.69 (95% CI: 0.59–0.79) for NCRT+NTT (**Figure 9D**).

#### 3.3.3 Sensitivity analysis and publication bias

Heterogeneity within each subgroup was assessed through sensitivity analyses. In the NCRT group, heterogeneity for TRG1 + 2 decreased notably after excluding the study by Reynolds et al. (29), yielding an ES of 0.62 (95% CI: 0.54–0.70; I²=70.73%). For the surgical resection rate, heterogeneity was eliminated (I²=0.00%, P = 0.76) after removing Stahl et al. (20), with a recalculated ES of 0.82 (95% CI: 0.80–0.85) using a fixed-effects model. Heterogeneity in surgical complications decreased after the exclusion of Tepper et al. (23), resulting in an ES of 0.55 (95% CI: 0.49–0.62; I²=56.09%). No significant changes in heterogeneity were observed in the remaining groups. Egger’s test and funnel plots (**Supplementary Table S2**, **Supplementary Figures S2–5**) indicated potential publication bias for R0 resection in the NCT group and grade ≥3 TRAEs in the NCIT group. No publication bias was detected in the other subgroups.

## 4 Discussion

The study is the first bibliometric analysis focused specifically on neoadjuvant therapy for esophageal cancer within our knowledge. In addition to employing traditional bibliometric methods, we systematically searched for and analyzed phase III RCTs in this field. This dual approach combines the strengths of bibliometric analysis—such as identifying publication trends, collaborative networks, and research hotspots (34)—with the systematic review of high-level clinical evidence, thereby addressing a common limitation of bibliometric studies, which often lack a detailed synthesis of clinical data.

### 4.1 Publication trends and research hotspots

Over the past 25 years, the volume of neoadjuvant therapy publications for esophageal cancer has increased significantly, with peaks observed in 2022 and 2024. These surges likely reflect the publication of pivotal clinical studies and the emergence of novel research directions. Indeed, from 2008 to 2016, with the reports of CROSS, FLOT4, CALGB 9781, and JCOG1109, chemotherapy and radiotherapy became the main clinical strategies for neoadjuvant treatment of esophageal cancer (5, 6, 8, 23). Recently, ICIs combined with chemotherapy, have demonstrated promising outcomes for non-small cell lung cancer in trials like CheckMate 816, AEGEAN, and KEYNOTE-671 (35–37). In 2024, this progress is further supported by studies, such as ESCORT-NEO/NCCES01 and HCHTOG 1909 (12, 30), which suggest that there might be clinical benefits from neoadjuvant chemoimmunotherapy for esophageal cancer. Therefore, over the past two decades, the neoadjuvant treatment strategy for esophageal cancer has evolved from chemotherapy and radiotherapy to immunotherapy, and from single treatment to diversified treatment.

The United States, as the country with the highest volume of publications, demonstrated a leading role and strong research capacity in the field, followed by China, Germany, Japan, and the Netherlands. Most of the countries with substantial research output were concentrated in the Americas, East Asia, and Europe—regions with higher incidence rates of ESCC or EAD (38). These regions also exhibited close international collaboration, further supporting their active engagement in advancing neoadjuvant therapy research. Similarly, most of the high-output institutions were based in these countries. Notably, Karolinska Institutet in Sweden has been identified as the institution with the highest volume of institutional publications, underscoring its prominent contributions and leadership in this research area.

In the analysis of cited authors, Ajani JA. exhibited high centrality, which is likely attributable to his contributions to the National Comprehensive Cancer Network (NCCN) clinical guidelines on neoadjuvant therapy for esophageal cancer (39, 40). Additionally, the CROSS study series (5, 9, 10) by van Hagen P, Shapiro Joel, and Eyck Ben M et al. stood out for their high citation frequencies and influence, reflecting their critical impact on shaping current treatment paradigms. This series of studies compared the median OS and 10-year follow-up data of neoadjuvant carboplatin + paclitaxel combined with radiotherapy versus surgery alone for locally advanced esophageal cancer, systematically and comprehensively demonstrating the clinical benefits of NCRT. Most of the other highly cited publications were phase III clinical trials on neoadjuvant therapy for esophageal or gastric cancer, such as FLOT4 (6) and NEOCRTEC5010 (24), or high-quality meta-analyses of major significance (41, 42). These studies consistently revealed that NCRT and NCT significantly improved patients’ disease-free survival (DFS) and OS. Furthermore, the CheckMate 577 trial evaluated postoperative adjuvant therapy with nivolumab in esophageal cancer, reporting a more prolonged DFS of 22.4 months (95% CI, 16.6 to 34.0) in the experimental group than that in the control group (hazard ratio [HR] 0.69; 96.4% CI, 0.56 to 0.86; P<0.001) (33). The rising citation frequency of this study indicates that NIT is increasingly becoming a focal point in esophageal cancer research (43–45), as further supported by recent phase III trials such as ESCORT-NEO/NCCES01 and HCHTOG 1909.

The analysis of keywords provides insight into research hotspots, evolving trends, and future directions within the field. In our study, the identified keywords were primarily associated with neoadjuvant treatment regimens, tumor histological types, tumor location, and follow-up outcomes. In 2009, Bollschweiler Elfriede reported notable discrepancies in the response to NCRT between ESCC and EAD (46). Consistently, the CROSS trial demonstrated a marked survival benefit for ESCC, reporting a median OS of 81.6 months (95% CI: 47.2–116.0) in the preoperative chemoradiotherapy group, compared to 43.2 months (95% CI: 24.9–61.4) for EAD (9)—nearly a twofold difference in median OS between the two histological subtypes. Additionally, the FLOT4 trial, which focused on gastroesophageal junction and gastric adenocarcinomas, demonstrated that fluorouracil, leucovorin, oxaliplatin, and docetaxel (FLOT) regimen resulted in significantly better pathological remission rates than those of the ECX or ECF regimens, which include epirubicin, cisplatin, and capecitabine or fluorouracil. Recent studies such as ESOPEC and Neo-AEGIS have further compared the CROSS regimen to preoperative chemotherapy alone in patients with EAD, aiming to refine treatment strategies based on histological and anatomical differences. In the Neo-AEGIS study, the 3-year OS rates were 55% (95% CI: 47–62) in the NCT group and 57% (95% CI: 49–64) in the CROSS group, with no statistically difference observed (HR: 1.03; 95% CI: 0.77–1.38; P = 0.82). Similarly, no statistically difference was found in median DFS (HR: 0.89; 95% CI: 0.68–1.17; P = 0.41). However, the CROSS regimen demonstrated significantly higher rates of pCR, MPR, and R0 resection than those of chemotherapy alone (29). In contrast, the ESOPEC trial, which employed the FLOT regimen for NCT, reported a significantly higher 3-year OS of 57.4% (95% CI: 50.1–64.0) compared to 50.7% (95% CI: 43.5–57.5) in the CROSS group (HR: 0.70; 95% CI: 0.53–0.92; P = 0.01) (11). The differing outcomes of these two trials may be attributed to variations in the chemotherapy regimens utilized. These findings underscore the importance of tailoring neoadjuvant treatment strategies for esophageal cancer based on histological subtypes, highlighting the need for personalized, pathology-specific approaches to optimize clinical outcomes.

Cluster analysis of the keywords identified several groups related to diagnostic methods and the evaluation of treatment efficacy, including PET, response prediction, and EUS. While numerous high-quality meta-analyses demonstrated that individual imaging modalities—such as computed tomography (CT), PET-CT, or EUS—have limited accuracy in predicting pathological responses to neoadjuvant therapy in esophageal cancer (47, 48), combining multiple imaging techniques or integrating imaging with clinical staging data may enhance decision-making (49, 50). Recent advancements in deep learning model development, incorporating tumor imaging, pathological features, and clinical variables has shown promise in accurately predicting pathological responses (51–53).

### 4.2 Efficacy and safety of neoadjuvant therapy

The efficacy and safety of neoadjuvant therapy for esophageal cancer were discussed according to different regimens. Our meta-analysis revealed that the NCRT group outperformed the NCT group in terms of pCR, TRG1, TRG1 + 2, and R0 resection rates. A recent meta-analysis confirmed that NCRT is associated with a higher pCR rate than that of the NCT group (54). However, this advantage did not consistently translate into improved OS (42). A phase III RCT further supported that progression-free survival (PFS) was significantly higher in the NCRT group than in the NCT group, whereas the discrepancy in OS between NCT and NCRT was not significant (P = 0.055) (22). The lack of OS benefit in the NCRT group may be partially attributed to malnutrition, which can reduce patient tolerance to TRAEs (55). For ESCC, the CMISG1701 study also reached a similar conclusion, and even did not demonstrated a significant difference in PFS among patients with cT3–4aN0–1M0 esophageal cancer (15). Recent studies have indicated that NCIT can significantly improve the PCR rate of ESCC patients compared with NCT. However, the EFS and OS are still immature (12). Short-term follow-up data from HCHTOG 1909 support the use of NCIT for ESCC, but long-term benefits need to be confirmed by follow-up data from phase III RCTs. For EAD, FLOT has long been the standard treatment protocol (6, 11), but there is significant potential for immunotherapy to change this situation. A phase II clinical trial in esophageal and gastric adenocarcinoma reported a pCR rate of 21.1% and an MPR rate of 44.7% (56). This means that compared to NCT, it has a higher pathological response rate, which is consistent with our results. Therefore, the optimal strategy of neoadjuvant therapy for esophageal cancer remains to be determined. NCIT is a potential optimal regimen, but follow-up data still need to be further studied. In addition, some studies have investigated the combination of NCRT with immunotherapy (NCRT+NIT). In some studies of pancreatic cancer, adding neoadjuvant immunotherapy is safe, but its efficacy is difficult to guarantee (57, 58). For esophageal cancer, no statistically significant pathological improvement was found with NCRT+NIT compared with NCRT (59, 60). However, a meta-analysis reported a greater increase in grade ≥3 TRAEs in the NCRT+NIT than that in the NCIT group (61), suggesting that the elevated toxicity may be attributable to the addition of radiotherapy. Evidence on NCRT+NTT remains limited. One study reported potential improvements in PFS and OS with this regimen (HR: 0.79; 95% CI: 0.58–1.07; P = 0.13; HR: 0.73; 95% CI: 0.52–1.01; P = 0.055, respectively) (14), and its clinical applicability requires further investigation.

Regarding the safety of the treatment, grade ≥3 TRAEs were significantly higher in the NCRT group than in the NCT and NCIT groups. These adverse events were primarily hematologic toxicities—such as leukopenia, neutropenia, and thrombocytopenia—as well as gastrointestinal complications, including anorexia, constipation, diarrhea, and vomiting. Esophageal-related complications, such as esophageal perforation and esophagitis, were also more frequent (5). The increased incidence of grade ≥3 TRAEs for NCRT may reflect the inherent toxicity of radiotherapy-based regimens (62, 63). Notably, a sub-study of the SAKK 75/08 trial found that NCRT significantly increased the risk of sarcopenia, which was associated with a higher incidence of grade ≥3 TRAEs; however, it did not result in increased postoperative mortality (64). However, it is important to note that only one study in the NCRT+NTT group reported grade ≥3 TRAEs, and further research is required to verify the reliability and universality of the result. In our meta-analysis, postoperative complication rates were comparable among all four treatment groups, though 30-day postoperative mortality was slightly higher in the NCRT and NCRT+NTT groups, which is consistent with the SAKK 75/08 sub-study (**Table 8**).

**Table 8:** Comparison of different neoadjuvant treatment regimens for esophageal cancer.

Treatment regimens	Advantages	Disadvantages
NCT	Lower grade≥3 TRAEs	Lower pCR, TRG1, TRG1 + 2, and R0 resection
NCRT	Higher pCR, TRG1, TRG1 + 2, and R0 resection	Higher grade≥3 TRAEs
NCIT	Higher pCR, TRG1, TRG1 + 2, and R0 resection Lower grade≥3 TRAEs	OS not reported
NCRT+NTT	Higher pCR, TRG1, and R0 resection	Higher grade≥3 TRAEs OS not reported

NCT, Neoadjuvant chemotherapy; NCIT, Neoadjuvant chemoimmunotherapy; NCRT, Neoadjuvant chemoradiotherapy; NTT, Neoadjuvant targeted therapy; OS, Overall Survival; pCR, complete pathologic response; TRAEs, treatment-related adverse events; TRG, Tumor regression grade.

### 4.3 Limitations

In this study, we conducted the first bibliometric analysis of neoadjuvant therapy for esophageal cancer along with a systematic review of relevant phase III RCTs. While the comparison of PFS and OS between NCT and NCRT has been explored in high-quality meta-analyses, follow-up data from the phase III RCTs investigating the NCIT and NCRT+NTT groups remain immature. Therefore, this study focused on summarizing and analyzing the ESs associated with these various treatment regimens.

Considerable heterogeneity was observed within the NCT and NCRT groups. Through sensitivity analysis, only the heterogeneity of the surgical resection rate in the NCRT group was significantly reduced, but the ES did not change significantly. This indicates that the data of this study is stable. The higher heterogeneity is probably related to the different pathological types, chemotherapy or radiotherapy regimens, treatment cycles, and clinical stage of the tumor, which limits the generalizability of this study to some extent. Therefore, clinicians should choose individualized treatment for different pathological types and clinical stages.

Furthermore, this study also has other limitations. First, the bibliometric analysis was solely based on English literature retrieved from the WoSCC, potentially excluding relevant studies indexed in other databases. To a certain degree, there is a possibility of selection bias, which limits the generalizability of the conclusion. Second, the number of studies included in the NCIT group and the NCRT+NTT group was limited, and the generalizability of the conclusions still requires further verification through more RCTs in the future. Third, a degree of publication bias was detected in a subset of the data, underscoring the requirement for further investigation to validate and refine these findings.

In conclusion, neoadjuvant therapy for esophageal cancer has significantly developed over the past 25 years. The efficacy and safety of NCRT and NCT have been well established, supported by robust long-term follow-up data. In contrast, NIT has demonstrated promising efficacy in recent years; however, it currently lacks validation from long-term follow-up data from phase III RCTs. Notably, the effectiveness of neoadjuvant treatment regimens appears to vary depending on tumor histology and clinical stage. Therefore, further high-quality studies are warranted to refine patient stratification and determine the most appropriate individualized neoadjuvant treatment strategies.

## Data Availability

The original contributions presented in the study are included in the article/**Supplementary Material**. Further inquiries can be directed to the corresponding author.
