# Supplementary material for: Research trends in neoadjuvant therapy for esophageal cancer: a bibliometric and meta-analysis
Source: Front Immunol. 2025 Nov 19;16:1646440. doi: 10.3389/fimmu.2025.1646440 (PMC12672519; doi:10.3389/fimmu.2025.1646440)
Supplement: Supplementary file 1 [file DataSheet1.doc]

Supplementary Material

# Supplementary Data

**Supplementary Table 1.** Studies data on neoadjuvant therapy for esophageal cancer.

**Supplementary Table 2.** *P* value of Subgroup Egger's test.

**Supplementary Figure 1.** Quality assessment of phase III RCT.

**Supplementary Figure 2.** Funnel plots of NCT group. **(A)** pCR. **(B)** TRG1. **(C)** TRG1+2. **(D)** R0 resection. **(E)** Surgical resection. **(F)** Surgical complications. **(G)** Postoperative 30 days of death. **(H)** TRAEs of grade ≥3.

**Supplementary Figure 3.** Funnel plots of NCRT group. **(A)** pCR. **(B)** TRG1. **(C)** TRG1+2. **(D)** R0 resection. **(E)** Surgical resection. **(F)** Surgical complications. **(G)** Postoperative 30 days of death. **(H)** TRAEs of grade ≥3.

**Supplementary Figure 4.** Funnel plots of NCIT group. **(A)** pCR. **(B)** TRG1. **(C)** TRG1+2. **(D)** R0 resection. **(E)** Surgical resection. **(F)** Surgical complications. **(G)** Postoperative 30 days of death. **(H)** TRAEs of grade ≥3.

**Supplementary Figure 5.** Funnel plots of NCRT+NTT group. **(A)** pCR. **(B)** TRG1. **(C)** R0 resection. **(D)** Surgical resection. **(E)** Postoperative 30 days of death. **(F)** TRAEs of grade ≥3.

**Search strategy**

# Supplementary Figures and Tables

| Study | Neoadjuvant treatment regimen | pCR | TRG1 | TRG1+2 | TRAEs  grade≥3 | Surgical resection | R0 resection | Surgical  complications | 30-day death |
| --- | --- | --- | --- | --- | --- | --- | --- | --- | --- |
| Kato K, 2024 | DDP+FU | 4 | NR | NR | NR | NR | 165 | NR | 2 |
|  | DDP+FU+DTX | 40 | NR | NR | NR | NR | 171 | NR | 0 |
|  | DDP+FU+41.4 Gy | 77 | NR | NR | NR | NR | 175 | NR | 1 |
| van Hagen P, 2012  Shapiro J, 2015  Eyck BM, 2021 | CBP+PTX+41.4 Gy | NR | 47 | 99 | NR | NR | 148 | NR | 10 |
| Hoeppner J, 2025 | FU+LV+OXP+DTX | 32 | 36 | 83 | 120 | 192 | 182 | NR | 2 |
|  | CBP+PTX+41.4 Gy | 18 | 24 | 95 | 98 | 179 | 172 | NR | 3 |
| Qin J, 2024 | Cam+nab-PTX+DDP | 37 | 47 | 71 | 45 | 114 | 113 | 39 | 1 |
|  | Cam+PTX+DDP | 20 | 23 | 44 | 38 | 116 | 111 | 45 | 2 |
|  | PTX+DDP | 6 | 7 | 19 | 36 | 103 | 95 | 33 | 1 |
| Lee JL, 2004 | DDP+FU+45.6 Gy | 15 | NR | NR | NR | NR | 35 | NR | 1 |
| Ruhstaller T, 2018 | DDP+DTX+CET+45 Gy | NR | 48 | NR | NR | 131 | 125 | NR | 4 |
|  | DDP+DTX+45 Gy | NR | 42 | NR | NR | 128 | 126 | NR | 4 |
| Tang H, 2023  Wang H, 2021 | PTX+DDP+40 Gy | 31 | 40 | 71 | NR | 112 | 109 | 54 | NR |
|  | PTX+DDP | 3 | 4 | 14 | NR | 104 | 100 | 46 | NR |
| Mariette C, 2014  Robb WB, 2015  Robb WB, 2025 | DDP+FU+45 Gy | 27 | 33 | 56 | NR | 81 | 76 | 45 | 6 |
| Stahl M, 2005 | FU+LV+ETO+DDP and ETO+DDP+40 Gy | 18 | NR | NR | NR | 55 | 51 | NR | NR |
| Stahl M, 2009  Stahl M, 2017 | FU+LV+DDP | 1 | NR | NR | NR | 49 | 41 | NR | NR |
|  | FU+LV+DDP and ETO+DDP+30 Gy | 7 | NR | NR | NR | 45 | 43 | NR | NR |
| Tepper J, 2008 | DDP+FU+50.4 Gy | 10 | NR | NR | 28 | 22 | NR | 24 | 0 |
| Yang H, 2018  Yang H, 2021 | NVB+DDP+40.0 Gy | 80 | NR | NR | NR | 184 | 182 | NR | 0 |
| Alderson D, 2017 | DDP+FU | NR | 5 | 12 | 73 | 387 | 212 | 224 | 10 |
|  | EPI+DDP+CAPE | NR | 21 | 37 | 108 | 364 | 223 | 233 | 11 |
| Burmeister BH, 2005 | DDP+FU+35 Gy | NR | 16 | NR | NR | 105 | 103 | 63 | NR |
| Safran HP, 2022 | PTX+CBP+TRA+50.4 Gy | 22 | NR | NR | 66/95 | 82 | 80 | NR | NR |
|  | PTX+CBP+50.4 Gy | 23 | NR | NR | 76 | 78 | 78 | NR | NR |
| Reynolds JV, 2023 | EPI+DDP/OXP+FU/CAPE or FU+OXP+LV+DTX | 7 | 8 | 19 | 77 | NR | 119 | 99 | NR |
|  | PTX+CBP+41.4 Gy | 20 | 23 | 64 | 65 | NR | 131 | 105 | NR |
| Zheng Y, 2024 | PTX+DDP+TOR | 19 | 31 | 45 | 15 | 102 | 102 | 99 | 2 |
|  | PTX+DDP | 4 | 6 | 13 | 16 | 88 | 88 | 87 | 0 |
| Noronha V, 2025 | DDP/CBP+FU | 14 | 16 | 25 | 124 | NR | 102 | NR | 4 |
|  | DDP/CBP+PTX | 28 | 33 | 40 | 97 | NR | 110 | NR | 4 |
| Ando N, 2012 | DDP+FU | NR | NR | NR | NR | 154 | 147 | NR | 1 |

**Supplementary Table 1.** Studies data on neoadjuvant therapy for esophageal cancer

**Supplementary Table 2.** *P* value of Subgroup Egger's test.

| Subgroups | pCR | TRG1 | TRG1+2 | TRAEs grade≥3 | Surgical resection | R0 resection | Surgical  complications | 30 days of death |
| --- | --- | --- | --- | --- | --- | --- | --- | --- |
| NCT | 0.231 | 0.341 | 0.311 | 0.230 | 0.576 | 0.026 | 0.989 | 0.108 |
| NCRT | 0.653 | 0.112 | 0.184 | 0.192 | 0.107 | 0.303 | 0.320 | 0.734 |
| NCIT | 0.812 | 0.921 | 0.914 | 0.046 | 0.463 | 0.474 | 0.124 | 0.628 |
| NCRT+NTT | NA | NA | NA | NA | NA | NA | NA | NA |


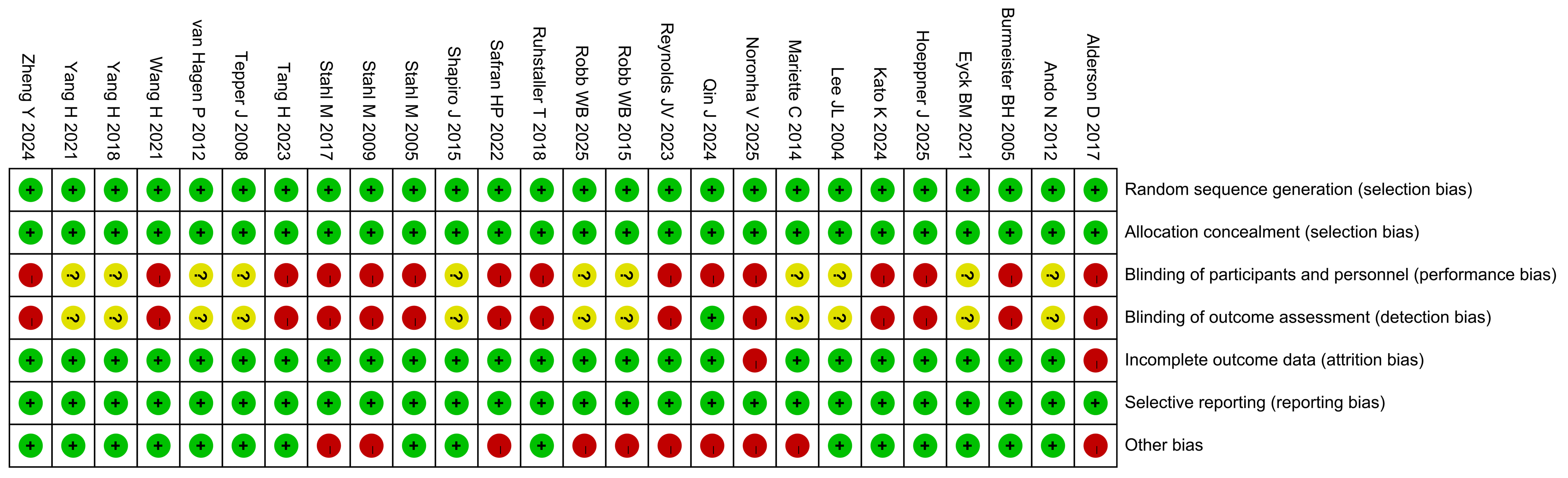
**Supplementary Figure 1.** Quality assessment of phase III RCT.


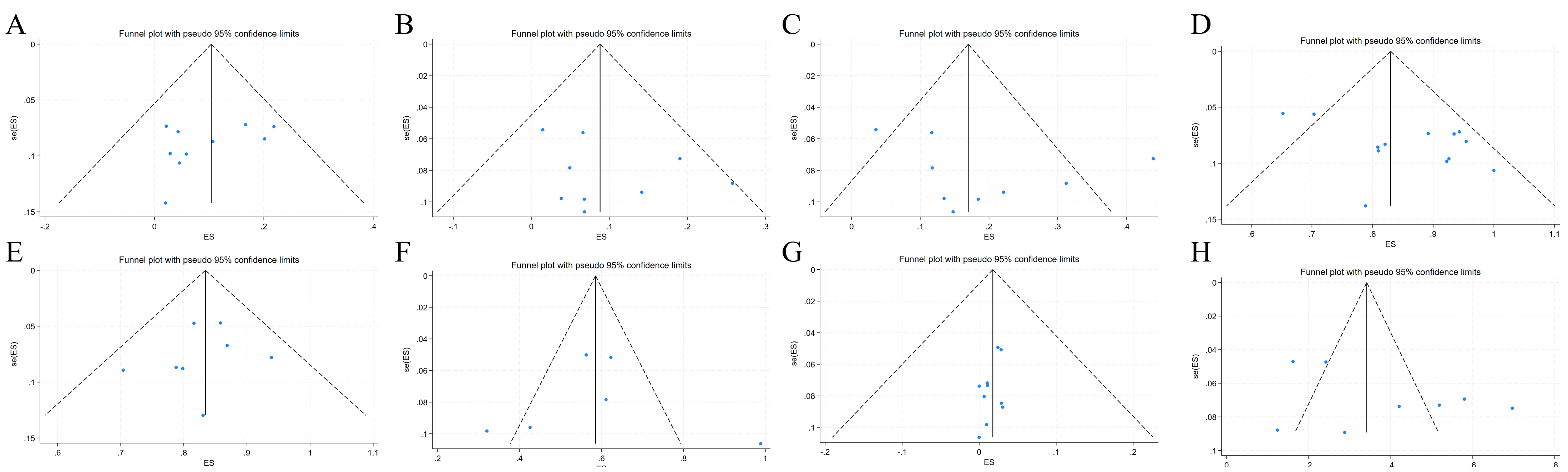


**Supplementary Figure 2.** Funnel plots of NCT group. **(A)** pCR. **(B)** TRG1. **(C)** TRG1+2. **(D)** R0 resection. **(E)** Surgical resection. **(F)** Surgical complications. **(G)** Postoperative 30 days of death. **(H)** TRAEs of grade ≥3.


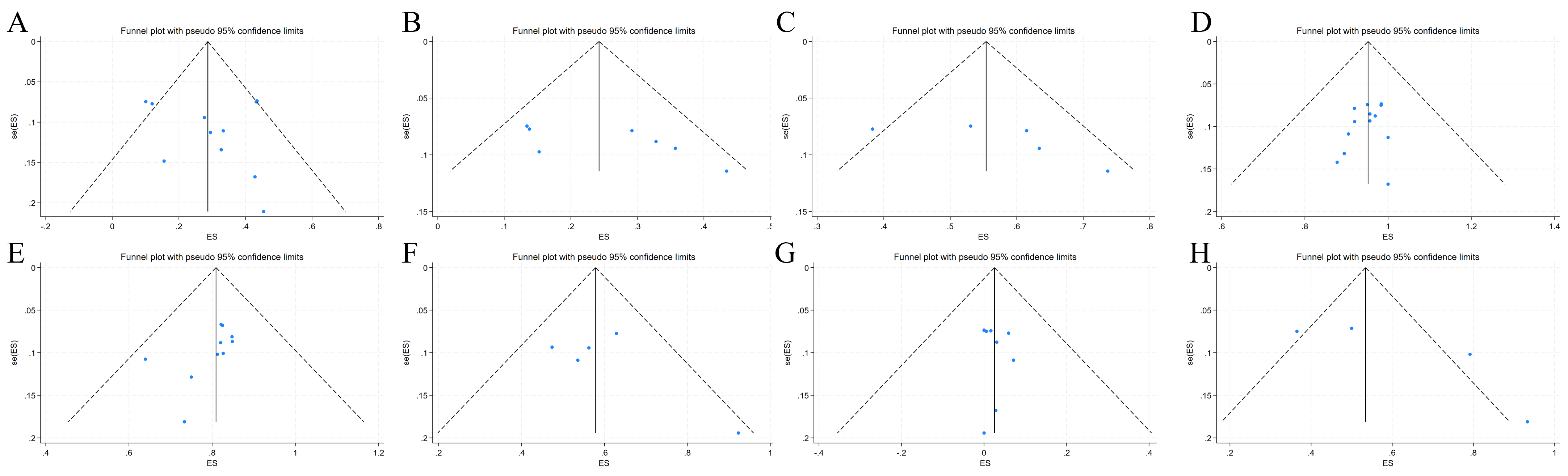


**Supplementary Figure 3.** Funnel plots of NCRT group. **(A)** pCR. **(B)** TRG1. **(C)** TRG1+2. **(D)** R0 resection. **(E)** Surgical resection. **(F)** Surgical complications. **(G)** Postoperative 30 days of death. **(H)** TRAEs of grade ≥3.


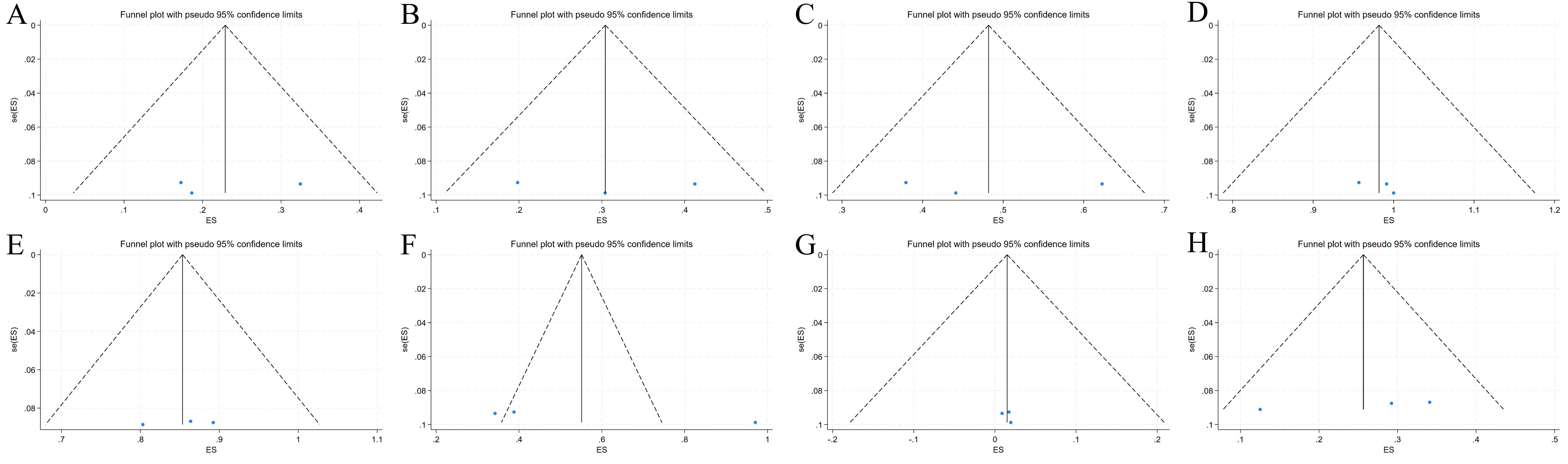


**Supplementary Figure 4.** Funnel plots of NCIT group. **(A)** pCR. **(B)** TRG1. **(C)** TRG1+2. **(D)** R0 resection. **(E)** Surgical resection. **(F)** Surgical complications. **(G)** Postoperative 30 days of death. **(H)** TRAEs of grade ≥3.


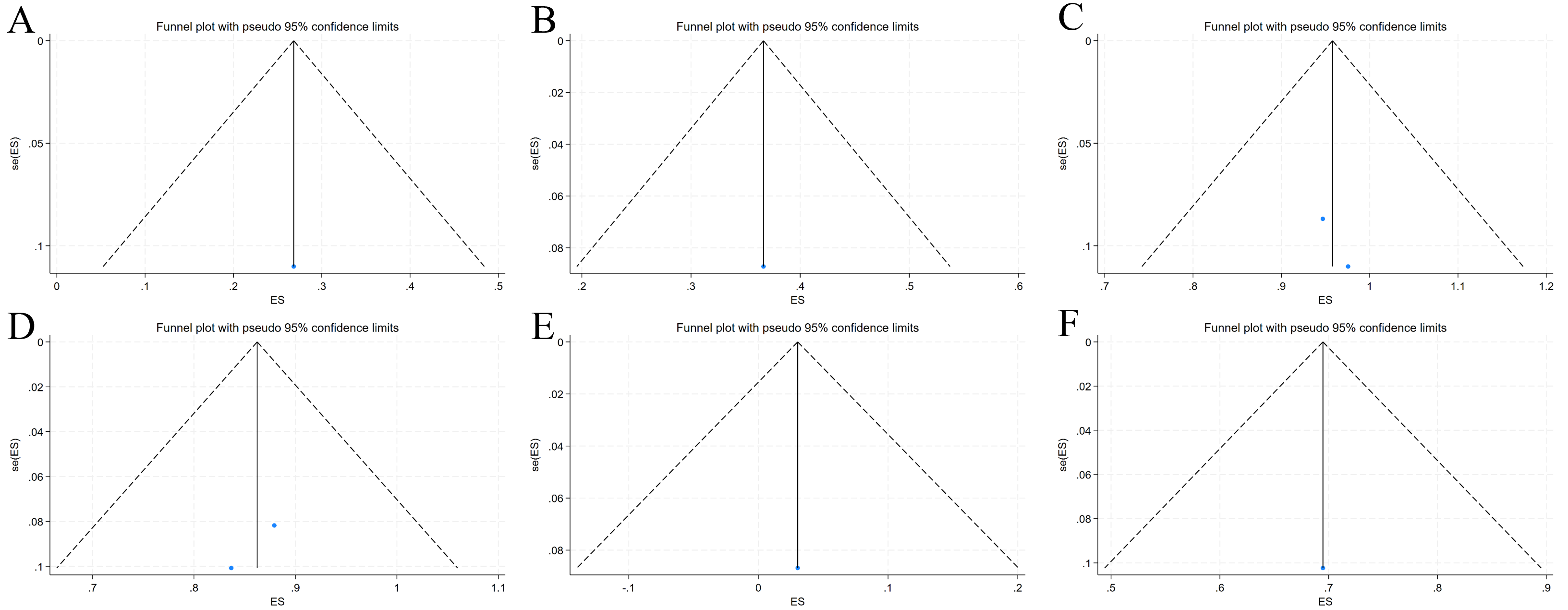


**Supplementary Figure 5.** Funnel plots of NCRT+NTT group. **(A)** pCR. **(B)** TRG1. **(C)** R0 resection. **(D)** Surgical resection. **(E)** Postoperative 30 days of death. **(F)** TRAEs of grade ≥3.

**Abbreviation:** AD, Adenosquamous carcinoma; BCC, Basal cell carcinoma; Cam, Camrelizumab; CAPE, Capecitabine; CBP, Carboplatin; CET, Cetuximab; DDP, Cisplatin; DTX, Docetaxel; EPI, Epirubicin; ETO, Etoposide; FU, Fluorouracil; ITT, Intention-to-treat population; LV, Leucovorin; nab-PTX, Nanoparticle Albumin-Bound Paclitaxel; NA, Not applicable; NCT, Neoadjuvant chemotherapy; NCIT, Neoadjuvant chemoimmunotherapy; NCRT, Neoadjuvant chemoradiotherapy; NR, Not reported; NTT, Neoadjuvant targeted therapy; NVB, Vinorelbine; OXP, Oxaliplatin; pCR, complete pathologic response; PTX, Paclitaxel; RCT, Randomized controlled trial; SCC, Squamous cell carcinoma; TOR, Toripalimab; TRA, Trastuzumab; TRAEs, treatment-related adverse events; TRG, Tumor regression grade.

# Search strategy

**PubMed**

(("Esophageal Neoplasms"[Mesh]) OR ((((Esophageal Neoplasm[Title/Abstract]) OR (Esophageal Cancer[Title/Abstract])) OR (Esophagus Cancer[Title/Abstract])) OR (Esophagus Neoplasm[Title/Abstract]))) AND ((RCT[Title/Abstract]) OR ("Randomized controlled trial"[Publication Type])) AND (("Neoadjuvant Therapy"[Mesh]) OR ((((((((((((((((((((((((((((((((((((((((((((((((Neoadjuvant Therapies[Title/Abstract]) OR (Therapy, Neoadjuvant[Title/Abstract])) OR (Neoadjuvant Treatment[Title/Abstract])) OR (Neoadjuvant Treatments[Title/Abstract])) OR (Treatment, Neoadjuvant[Title/Abstract])) OR (Neoadjuvant Radiotherapy[Title/Abstract])) OR (Neoadjuvant Radiotherapies[Title/Abstract])) OR (Radiotherapy, Neoadjuvant[Title/Abstract])) OR (Neoadjuvant Radiation Treatment[Title/Abstract])) OR (Neoadjuvant Radiation Treatments[Title/Abstract])) OR (Radiation Treatment, Neoadjuvant[Title/Abstract])) OR (Treatment, Neoadjuvant Radiation[Title/Abstract])) OR (Neoadjuvant Radiation Therapy[Title/Abstract])) OR (Neoadjuvant Radiation Therapies[Title/Abstract])) OR (Radiation Therapy, Neoadjuvant[Title/Abstract])) OR (Therapy, Neoadjuvant Radiation[Title/Abstract])) OR (Neoadjuvant Radiation[Title/Abstract])) OR (Neoadjuvant Radiations[Title/Abstract])) OR (Radiation, Neoadjuvant[Title/Abstract])) OR (Neoadjuvant Systemic Therapy[Title/Abstract])) OR (Neoadjuvant Systemic Therapies[Title/Abstract])) OR (Systemic Therapy, Neoadjuvant[Title/Abstract])) OR (Therapy, Neoadjuvant Systemic[Title/Abstract])) OR (Neoadjuvant Systemic Treatment[Title/Abstract])) OR (Neoadjuvant Systemic Treatments[Title/Abstract])) OR (Systemic Treatment, Neoadjuvant[Title/Abstract])) OR (Treatment, Neoadjuvant Systemic[Title/Abstract])) OR (Neoadjuvant Chemotherapy[Title/Abstract])) OR (Chemotherapy, Neoadjuvant[Title/Abstract])) OR (Neoadjuvant Chemotherapies[Title/Abstract])) OR (Neoadjuvant Chemotherapy Treatment[Title/Abstract])) OR (Chemotherapy Treatment, Neoadjuvant[Title/Abstract])) OR (Neoadjuvant Chemotherapy Treatments[Title/Abstract])) OR (Treatment, Neoadjuvant Chemotherapy[Title/Abstract])) OR (Neoadjuvant Chemoradiotherapy[Title/Abstract])) OR (Chemoradiotherapy, Neoadjuvant[Title/Abstract])) OR (Neoadjuvant Chemoradiotherapies[Title/Abstract])) OR (Neoadjuvant Chemoradiation Therapy[Title/Abstract])) OR (Chemoradiation Therapy, Neoadjuvant[Title/Abstract])) OR (Neoadjuvant Chemoradiation Therapies[Title/Abstract])) OR (Therapy, Neoadjuvant Chemoradiation[Title/Abstract])) OR (Neoadjuvant Chemoradiation Treatment[Title/Abstract])) OR (Chemoradiation Treatment, Neoadjuvant[Title/Abstract])) OR (Neoadjuvant Chemoradiation Treatments[Title/Abstract])) OR (Treatment, Neoadjuvant Chemoradiation[Title/Abstract])) OR (Neoadjuvant Chemoradiation[Title/Abstract])) OR (Chemoradiation, Neoadjuvant[Title/Abstract])) OR (Neoadjuvant Chemoradiations[Title/Abstract])))

**Embase**

#10 #3 AND #6 AND #9

#9 #7 OR #8

#8 'RCT':ab,ti

#7 'Randomized controlled trial'/exp

#6 #4 OR #5

#5 ‘neoadjuvant therapies’:ab,ti OR ‘therapy, neoadjuvant’:ab,ti OR ‘neoadjuvant treatment’:ab,ti OR ‘neoadjuvant treatments’:ab,ti OR ‘treatment, neoadjuvant’:ab,ti OR ‘neoadjuvant radiotherapy’:ab,ti OR ‘radiotherapy, neoadjuvant’:ab,ti OR ‘neoadjuvant radiation treatments’:ab,ti OR ‘neoadjuvant radiotherapies’:ab,ti OR ‘neoadjuvant radiation treatment’:ab,ti OR ‘radiation treatment, neoadjuvant’:ab,ti OR ‘treatment, neoadjuvant radiation’:ab,ti OR ‘neoadjuvant radiation therapy’:ab,ti OR ‘radiation therapy, neoadjuvant’:ab,ti OR ‘neoadjuvant radiation therapies’:ab,ti OR ‘therapy, neoadjuvant radiation’:ab,ti OR ‘neoadjuvant radiation’:ab,ti OR ‘neoadjuvant radiations’:ab,ti OR ‘radiation, neoadjuvant’:ab,ti OR ‘neoadjuvant systemic therapy’:ab,ti OR ‘systemic therapy, neoadjuvant’:ab,ti OR ‘therapy, neoadjuvant systemic’:ab,ti OR ‘neoadjuvant systemic therapies’:ab,ti OR ‘neoadjuvant systemic treatments’:ab,ti OR ‘neoadjuvant systemic treatment’:ab,ti OR ‘systemic treatment, neoadjuvant’:ab,ti OR ‘treatment, neoadjuvant systemic’:ab,ti OR ‘neoadjuvant chemotherapy’:ab,ti OR ‘chemotherapy, neoadjuvant’:ab,ti OR ‘neoadjuvant chemotherapies’:ab,ti OR ‘neoadjuvant chemotherapy treatment’:ab,ti OR ‘chemotherapy treatment, neoadjuvant’:ab,ti OR ‘neoadjuvant chemotherapy treatments’:ab,ti OR ‘treatment, neoadjuvant chemotherapy’:ab,ti OR ‘neoadjuvant chemoradiotherapy’:ab,ti OR ‘chemoradiotherapy, neoadjuvant’:ab,ti OR ‘neoadjuvant chemoradiotherapies’:ab,ti OR ‘neoadjuvant chemoradiation therapy’:ab,ti OR ‘chemoradiation therapy, neoadjuvant’:ab,ti OR ‘neoadjuvant chemoradiation therapies’:ab,ti OR ‘neoadjuvant chemoradiation treatment’:ab,ti OR ‘therapy, neoadjuvant chemoradiation’:ab,ti OR ‘neoadjuvant chemoradiation treatments’:ab,ti OR ‘chemoradiation treatment, neoadjuvant’:ab,ti OR ‘treatment, neoadjuvant chemoradiation’:ab,ti OR ‘neoadjuvant chemoradiation’:ab,ti OR ‘chemoradiation, neoadjuvant’:ab,ti OR ‘neoadjuvant chemoradiations’:ab,ti

#4 'neoadjuvant therapy'/exp

#3 #1 OR #2

#2 'Esophageal Neoplasm':ab,ti OR 'Esophageal Cancer':ab,ti OR 'Esophagus Neoplasm':ab,ti OR ' Esophagus Cancer':ab,ti

#1 'Esophageal Neoplasms'/exp

**Cochrane Library**

#1 Esophageal Neoplasms

#2 (Esophageal Neoplasm):ti,ab,kw OR (Esophageal Cancer):ti,ab,kw OR (Esophagus Cancer):ti,ab,kw OR (Esophagus Neoplasm):ti,ab,kw

#3 #1 or #2

#4 Neoadjuvant Therapy

#5 (Neoadjuvant Therapies ):ti,ab,kw OR (Therapy, Neoadjuvant):ti,ab,kw OR (Neoadjuvant Treatment):ti,ab,kw OR (Neoadjuvant Treatments):ti,ab,kw OR (Treatment, Neoadjuvant):ti,ab,kw OR (Neoadjuvant Radiotherapy):ti,ab,kw OR (Neoadjuvant Radiotherapies):ti,ab,kw OR (Radiotherapy, Neoadjuvant):ti,ab,kw OR (Neoadjuvant Radiation Treatment):ti,ab,kw OR (Neoadjuvant Radiation Treatments):ti,ab,kw OR (Radiation Treatment, Neoadjuvant):ti,ab,kw OR (Treatment, Neoadjuvant Radiation):ti,ab,kw OR (Neoadjuvant Radiation Therapy):ti,ab,kw OR (Neoadjuvant Radiation Therapies):ti,ab,kw OR (Radiation Therapy, Neoadjuvant):ti,ab,kw OR (Therapy, Neoadjuvant Radiation):ti,ab,kw OR (Neoadjuvant Radiation):ti,ab,kw OR (Neoadjuvant Radiations):ti,ab,kw OR (Radiation, Neoadjuvant):ti,ab,kw OR (Neoadjuvant Systemic Therapy):ti,ab,kw OR (Neoadjuvant Systemic Therapies):ti,ab,kw OR (Systemic Therapy, Neoadjuvant):ti,ab,kw OR (Therapy, Neoadjuvant Systemic):ti,ab,kw OR (Neoadjuvant Systemic Treatment):ti,ab,kw OR (Neoadjuvant Systemic Treatments):ti,ab,kw OR (Systemic Treatment, Neoadjuvant):ti,ab,kw OR (Treatment, Neoadjuvant Systemic):ti,ab,kw OR (Neoadjuvant Chemotherapy):ti,ab,kw OR (Chemotherapy, Neoadjuvant):ti,ab,kw OR (Neoadjuvant Chemotherapies):ti,ab,kw OR (Neoadjuvant Chemotherapy Treatment):ti,ab,kw OR (Chemotherapy Treatment, Neoadjuvant):ti,ab,kw OR (Neoadjuvant Chemotherapy Treatments):ti,ab,kw OR (Treatment, Neoadjuvant Chemotherapy):ti,ab,kw OR (Neoadjuvant Chemoradiotherapy):ti,ab,kw OR (Chemoradiotherapy, Neoadjuvant):ti,ab,kw OR (Neoadjuvant Chemoradiotherapies):ti,ab,kw OR (Neoadjuvant Chemoradiation Therapy):ti,ab,kw OR (Chemoradiation Therapy, Neoadjuvant):ti,ab,kw OR (Neoadjuvant Chemoradiation Therapies):ti,ab,kw OR (Therapy, Neoadjuvant Chemoradiation):ti,ab,kw OR (Neoadjuvant Chemoradiation Treatment):ti,ab,kw OR (Chemoradiation Treatment, Neoadjuvant):ti,ab,kw OR (Neoadjuvant Chemoradiation Treatments):ti,ab,kw OR (Treatment, Neoadjuvant Chemoradiation):ti,ab,kw OR (Neoadjuvant Chemoradiation):ti,ab,kw OR (Chemoradiation, Neoadjuvant):ti,ab,kw OR (Neoadjuvant Chemoradiations):ti,ab,kw

#6 #4 or #5

#7 Randomized controlled trial

#8 (RCT):ti,ab,kw

#9 #7 or #8

#10 #3 and #6 and #9

**Web of Science**

#4 3 AND #2 AND #1

#3 TS=(Randomized controlled trial or RCT)

#2 TS=(Neoadjuvant Therapy or Neoadjuvant Therapies or Therapy, Neoadjuvant or Neoadjuvant Treatment or Neoadjuvant Treatments or Treatment, Neoadjuvant or Neoadjuvant Radiotherapy or Neoadjuvant Radiotherapies or Radiotherapy, Neoadjuvant or Neoadjuvant Radiation Treatment or Neoadjuvant Radiation Treatments or Radiation Treatment, Neoadjuvant or Treatment, Neoadjuvant Radiation or Neoadjuvant Radiation Therapy or Neoadjuvant Radiation Therapies or Radiation Therapy, Neoadjuvant or Therapy, Neoadjuvant Radiation or Neoadjuvant Radiation or Neoadjuvant Radiations or Radiation, Neoadjuvant or Neoadjuvant Systemic Therapy or Neoadjuvant Systemic Therapies or Systemic Therapy, Neoadjuvant or Therapy, Neoadjuvant Systemic or Neoadjuvant Systemic Treatment or Neoadjuvant Systemic Treatments or Systemic Treatment, Neoadjuvant or Treatment, Neoadjuvant Systemic or Neoadjuvant Chemotherapy or Chemotherapy, Neoadjuvant or Neoadjuvant Chemotherapies or Neoadjuvant Chemotherapy Treatment or Chemotherapy Treatment, Neoadjuvant or Neoadjuvant Chemotherapy Treatments or Treatment, Neoadjuvant Chemotherapy or Neoadjuvant Chemoradiotherapy or Chemoradiotherapy, Neoadjuvant or Neoadjuvant Chemoradiotherapies or Neoadjuvant Chemoradiation Therapy or Chemoradiation Therapy, Neoadjuvant or Neoadjuvant Chemoradiation Therapies or Therapy, Neoadjuvant Chemoradiation or Neoadjuvant Chemoradiation Treatment or Chemoradiation Treatment, Neoadjuvant or Neoadjuvant Chemoradiation Treatments or Treatment, Neoadjuvant Chemoradiation or Neoadjuvant Chemoradiation or Chemoradiation, Neoadjuvant or Neoadjuvant Chemoradiations)

#1 TS=("Esophageal Neoplasms" OR "Esophageal Neoplasm" OR "Esophagus Neoplasm" OR "Esophageal Cancer" OR "Esophagus Cancer")
